# The stratification and prognostic importance of molecular and immune landscapes in clear cell renal cell carcinoma

**DOI:** 10.3389/fonc.2023.1256720

**Published:** 2023-10-02

**Authors:** Xinyu Zhai, Xinglin Chen, Jianyi Gu, Dongdong Guo, Xiangyang Zhan, Mingyue Tan, Dongliang Xu

**Affiliations:** Urology Centre, Shuguang Hospital Affiliated to Shanghai University of Traditional Chinese Medicine, Shanghai, China

**Keywords:** clear cell renal cell carcinoma, prognostic model, immune evasion, genetic alterations, DKK1

## Abstract

The aim of our research is to explore the various characteristics and genetic profiles of clear cell renal cell carcinoma (ccRCC) in order to discover possible predictors of prognosis and targets for treatment. By utilizing ssGSEA scores, we categorized patients with ccRCC into groups based on their phenotype, distinguishing between low and high. This categorization revealed significant variations in the expression of crucial immune checkpoint genes and Human Leukocyte Antigen (HLA) genes, suggesting the presence of a potential immune evasion tactic in different subtypes of ccRCC. A predictive model was built using genes that are expressed differently and linked to cell death, showing strong effectiveness in categorizing patient risk. Furthermore, we discovered a noteworthy correlation among risk scores, infiltration of immune cells, the expression of genes related to immune checkpoint inhibitors, and diverse clinical features. This indicates that our scoring system for risk could function as a comprehensive gauge of the severity of the disease. The examination of the mutational terrain further highlighted the predominance of particular genetic changes, including VHL and PBRM1 missense mutations. Finally, we have discovered the function of DKK1 in facilitating cell death in ccRCC, presenting an additional possibility for therapeutic intervention. The results of our study suggest the possibility of incorporating molecular information into clinical prediction, which could lead to personalized treatment approaches in ccRCC.

## Introduction

Annually, the global incidence of renal cell carcinoma (RCC) exceeds 400,000 cases. The average age of diagnosis is approximately 60 years old, with men being twice as prone to receiving this diagnosis compared to women (1). Around 70% of people diagnosed with clear cell RCC (ccRCC) possess one of the subtypes of RCC (2). Despite the potential for early identification and effective management of ccRCC using surgical or ablative approaches, approximately one-third of individuals will experience the spread of cancer to other parts of the body (3). Consequently, ccRCC is nearly universally fatal, and this distinction holds significant importance. Around 30% of patients with RCC have metastatic disease when they are first diagnosed (4). Recurrence is experienced by approximately 30% of patients who have their primary tumor completely removed. Between 10 and 25 percent of individuals with localized illness encounter relapse despite accidental identification and fully surgical removal (5). Currently, chemotherapy and radiation therapy are ineffective in treating all types of RCC (6). During the initial phase of the investigation, alternative treatment approaches were explored due to the insufficient effectiveness of chemotherapy and radiation therapy.

At present, there is no genetic biomarker that can be utilized as a dependable indicator of ccRCC prognosis or therapy. Traditional cytotoxic chemotherapeutics are typically ineffective in treating ccRCC (7). Previously, individuals diagnosed with metastatic RCC received cytokine therapy as a treatment option prior to the introduction of small molecule targeted therapies and immune checkpoint inhibitors (8). Response rates, however, remained low. Since their introduction in 2005 (9), anti-angiogenic small molecule tyrosine kinase inhibitors (TKIs) that primarily target VEGF signaling pathways have played a crucial role in therapy. Among the small molecule inhibitors approved in clinical trials (10), there are axitinib, cabozantinib, lenvatinib, pazopanib, sorafenib, and sunitinib. Checkpoint inhibitors that target CTLA-4, PD-1, and PD-L1 are used to kill cancer cells by restoring the adaptive immune system’s ability to target and eliminate them (11). Nivolumab and pembrolizumab inhibit the negative effect of T-cell PD-1 in conjunction with ipilimumab, whereas nivolumab inhibits CTLA-4 (12). Initially, the utilization of nivolumab in the second stage of treatment for therapies blocking checkpoints led to an enhancement in the survival of patients. It is crucial to highlight, though, that a considerable amount of individuals still succumb to the illness due to their immune systems’ resistance against checkpoint inhibitors, be it inherent or acquired (13). Hence, it is imperative to devise novel therapeutic protocols in order to enhance the effectiveness of ccRCC (14).

Over the last few years, with the progress of bioinformatics analysis in diverse domains, there has been a growing focus on the importance of biomarkers in predicting the outcome of human diseases. However, the study is limited in its focus, specifically examining the relationship between predictive factors and ccRCC. Therefore, our goal is to examine the potential markers for individuals with ccRCC, which could improve the detection and treatment of ccRCC. Furthermore, various algorithms were utilized to assess the impact of immunotherapy in patients with ccRCC. Additionally, the analysis of pathway enrichment was conducted to further identify the crucial pathways in patients with ccRCC and immunotherapy.

## Methods

### Data obtaining

We have successfully acquired RNAseq data and relevant clinical information for tumors in ccRCC patients from the TCGA dataset (https://portal.gdc.com). During our investigation, we utilized the Cancer Single-cell State Atlas (CancerSEA) database, a crucial and all-encompassing source that offers a functional atlas of single-cell states for diverse cancer categories. CancerSEA stands out for its capacity to decipher functional conditions from a cellular viewpoint, providing understanding into a wide array of functional states of cancer cells, encompassing growth, cell death, oxygen deficiency, blood vessel formation, infiltration, spreading, and more. By gathering single-cell transcriptome data from publicly available databases, users can investigate the expression of their genes of interest across different functional states and types of cancer.

### The analysis of gene expression differences

At first, the RNA-Seq data underwent data normalization to manage technical variability. The utilization of limma’s linear modeling and empirical Bayes moderation is well-suited for analyzing differential expression in RNA-Seq. The limma package in R was used to perform differential gene expression analysis, comparing ccRCC tumor samples with non-tumor samples. Differentially expressed genes (DEGs) were determined by analyzing their log_2_fold-change and adjusted p-value. We classified genes as significantly differentially expressed if their absolute log2 fold-change was greater than 1 and their adjusted p-value was less than 0.05.

### Developing the prognostic forecasting model in the ccRCC group

To begin with, we conducted a Cox regression analysis on a single variable to determine the genes that showed a significant correlation with the overall duration of survival. Genes that had a p-value lower than 0.05 were considered statistically significant and chosen for additional examination. Afterwards, we utilized the LASSO regression analysis to further refine our choice of genes. By minimizing the total of the absolute coefficients, this approach effectively causes certain coefficients to shrink to zero, thus functioning as a method for selecting features. To find the lambda value that minimized the mean cross-validated error, we employed ten-fold cross-validation. Next, the genes that were not eliminated by the LASSO regression analysis were included in a multivariate Cox regression analysis. The objective of this analysis was to construct a predictive model by identifying the autonomous predictive genes while accounting for other factors. We calculated the hazard ratios (HR) and their corresponding 95% confidence intervals (CI). The predictive model was created using the linear sum of the levels of gene expression, weighted by their corresponding coefficients obtained from the multivariate Cox regression analysis. Based on the risk score median, we categorized patients into groups of high-risk and low-risk.

### Enrichment analysis of gene sets using single sample

To obtain the enrichment scores of predetermined gene sets in each sample, we utilized ssGSEA, which is an expansion of GSEA, employing a single-sample approach. The ssGSEA method enables the characterization of individual patient’s gene set enrichment profile, providing an individualized and comprehensive representation of their biological states. The GSVA package in R was utilized to conduct ssGSEA. ssGSEA calculates an enrichment score for each sample, which indicates the extent to which genes in a specific gene set are collectively upregulated or downregulated in that sample. The ssGSEA score is determined by subtracting the weighted empirical cumulative distribution functions of the genes in the gene set from those of the remaining genes. Afterwards, the scores of enrichment are adjusted to fall within the range of 0 to 1.

### Multivariate and univariate Cox regression

In our study, we examined the prognosis of patients with ccRCC by analyzing the independent connections between gene expression, age, gender, grade, stage, T status, M status, and N status using both multivariate and univariate Cox regression analysis. Using the TCGA database, we calculated hazard ratios (HRs) and determined 95% confidence intervals (CIs). The survival ROC R package was employed to generate a time-varying ROC curve for Receiver Operating Characteristic.

### Pathway enrichment analysis

The relationship between the level of gene expression and the TCGA database in ccRCC samples was examined by analyzing the gene expression using the R packages ‘limma’ and ‘ggplot’ for gene analysis. We assessed the potential functions of important genes in ccRCC by analyzing KEGG and GO databases, using the R packages ‘ClusterProfiler’ and ‘org.Hs.eg.db’.

### Analysis of infiltration by immune cells

The RNA-Seq data was analyzed using CIBERSORT, an algorithm for deconvolution, to estimate the ratios of 22 phenotypes of human immune cells, which encompass B cells, T cells, natural killer cells, macrophages, dendritic cells, and various others. By utilizing a collection of reference gene expression values, known as a ‘signature matrix’, the algorithm can estimate the existence of various cell types by analyzing the gene expression data obtained from the bulk tumor sample. The results from the CIBERSORT analysis were further evaluated to determine the associations between immune cell proportions in each sample and clinical characteristics as well as outcomes.

### Somatic mutation analysis

Data on genetic changes in body cells were acquired from The Cancer Genome Atlas (TCGA) repository. The TCGA is an extensive database that offers multidimensional charts of significant genetic alterations in different forms of cancer. Our study specifically targeted the somatic mutation data obtained through whole-exome sequencing. The MAF files that were downloaded have been processed and analyzed using the R package called ‘maftools’. The main purpose of this package is to specifically create a tool for in-depth examination, depiction, and condensation of MAF files obtained from extensive sequencing investigations. In R, we utilized the `read.maf` function to import the MAF files. Additionally, we employed the `oncoplot` function to display the mutation landscape, which showcases the most frequently mutated genes and their corresponding mutation types. Additional plots were generated to gain a deeper comprehension of the genomic structure and the mutational patterns of the examined samples.

### Correlation analysis

We used either Pearson’s correlation coefficient or Spearman’s rank correlation coefficient, depending on the characteristics of the variables and the distribution of the data. The Pearson correlation coefficient was employed to assess the linear association between two datasets when they follow a normal distribution and consist of continuous variables. In situations where the data did not follow a normal distribution or if there was a non-linear relationship between variables, Spearman’s rank correlation coefficient was used to assess the monotonic relationship between the two datasets. The `cor.test()` function in R was used to calculate correlation coefficients, which yield both the correlation coefficient and its corresponding p-value. The range of the correlation coefficient (r) is -1 to 1. A value of -1 signifies a complete negative linear relationship, while a value of 1 signifies a complete positive linear relationship.

### Cell culture

Cell lines of renal cell carcinoma in humans (786-O, Caki-1) were grown in RPMI 1640 medium and DMEM, which were enriched with 10% fetal bovine serum (FBS; Gibco), 100 U/mL penicillin, and 100 μg/mL streptomycin (Gibco). The cell lines were cultured at a temperature of 37°C in a moist environment containing 5% carbon dioxide.

### Flow cytometry can be used to identify apoptosis

Cell apoptosis was evaluated by employing an Annexin V-FITC/PI Apoptosis Detection Kit in accordance with the guidelines provided by the manufacturer. By utilizing this technique, it becomes possible to measure the amount of cells undergoing early apoptosis (Annexin V+/PI-) and late apoptosis (Annexin V+/PI+), in addition to viable cells (Annexin V-/PI-) and necrotic cells (Annexin V-/PI+).

### Statistical analysis

Statistical analyses were performed using R software. A p-value < 0.05 was considered statistically significant.

## Results

### Identifying various phenotypes within the ccRCC cohort

To investigate the potential phenotypes associated with ccRCC cohort, the CancerSEA online dataset was utilized for exploration. The RCC cohort in CancerSEA was utilized to acquire various genes associated with multiple phenotypes, including angiogenesis, apoptosis, cell cycle, differentiation, DNA damage, DNA repair, EMT, hypoxia, inflammation, invasion, metastasis, proliferation, quiescence, and stemness. The CancerSEA provided the genes associated with each phenotype.

### The ssGSEA analysis revealed the relative scores of each phenotype in ccRCC cohort

**Figure 1:**
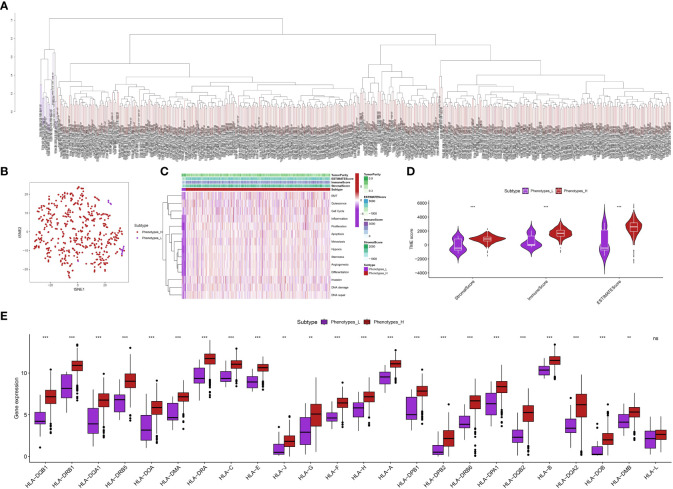
**(A)** The ssGSEA analysis revealed the patients with ccRCC in different groups; **(B)** The tSNE analysis showed the distribution of ccRCC patients with the phenotype-related scores; **(C)** The heatmap demonstrated the multiple phenotype-related scores in ccRCC cohort; **(D)** The correlation between TME and phenotypes-related scores; **(E)** The correlation between HLA-related genes and phenotype-related scores in ccRCC cohort. **p < 0.01; ***p < 0.001.

**Figure 2:**
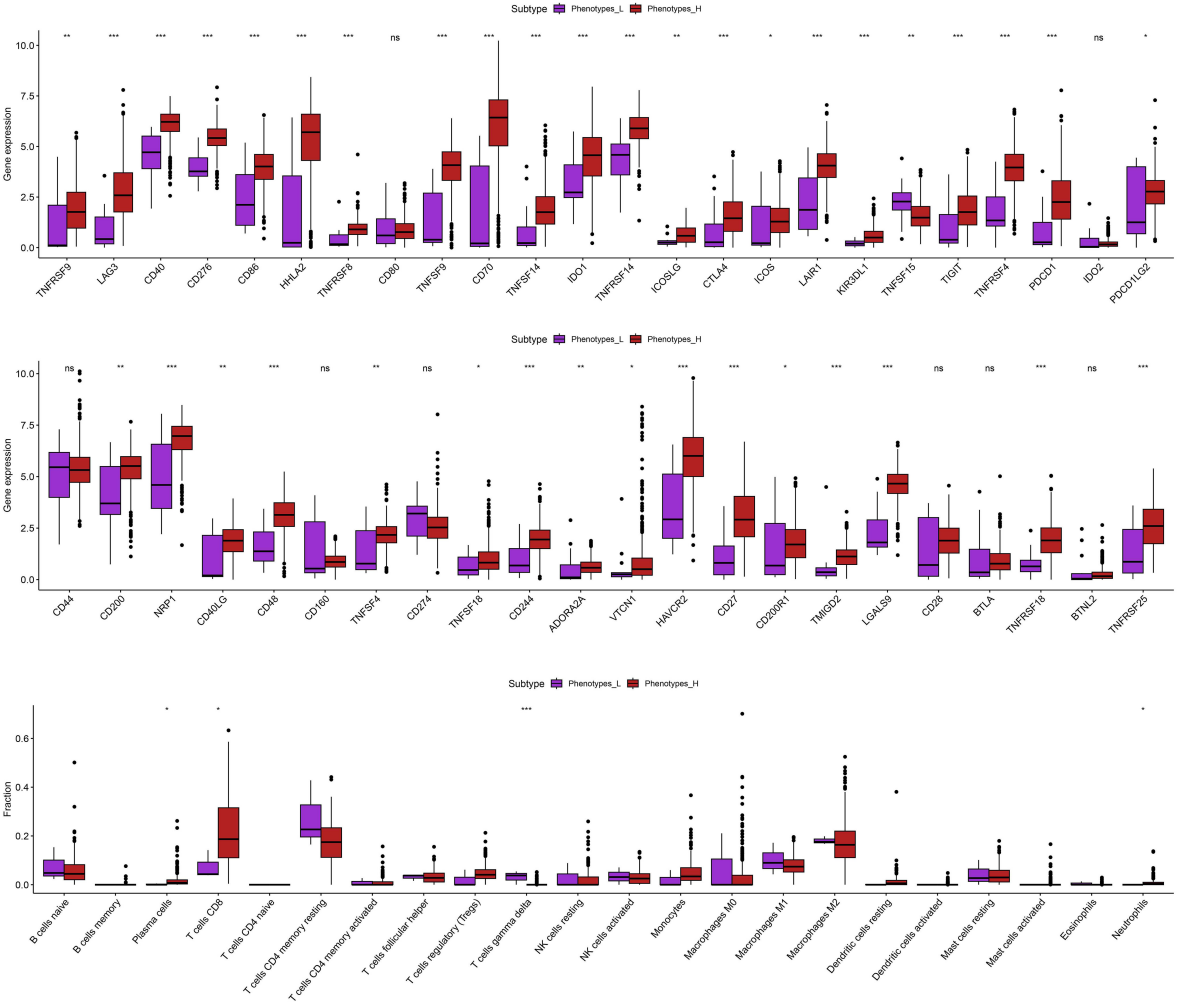
**(A, B)** The correlation between phenotypes-related score and the expression level of immune checkpoint-related scores; **(C)** The correlation between immune cell infiltration and phenotypes-related scores. *p < 0.05; **p < 0.01; ***p < 0.001. ns, not significant.

Using the ssGSEA analysis, we were able to acquire the relative scores for various phenotypes in ccRCC patients (**Figure 1A**). Next, we applied t-SNE, a nonlinear method for reducing dimensionality, to display the ssGSEA scores, which were originally in a high-dimensional space, in a two-dimensional space. The t-SNE analysis revealed clear clusters among the samples, indicating distinct gene set activity patterns (**Figure 1B**). Our samples exhibited distinct biological conditions or subtypes, as indicated by each cluster’s representation of a unique gene expression profile. In order to further examine these patterns, we created a heat map illustrating the ssGSEA scores. The heatmap indicated that the ccRCC individuals could be categorized into groups associated with low and high phenotypes (**Figure 1C**). Furthermore, we assess the association between low- and high phenotypes groups by considering the immune microenvironment (TME). The findings indicated that patients with elevated phenotypes in ccRCC also exhibited increased stromal score, immune score, and estimate score (**Figure 1D**). Following that, our examination concentrated on the levels of gene expression associated with Human Leukocyte Antigen (HLA), which is a vital element of the immune system’s reaction, among various cohorts in our investigation (**Figure 1E**). Significant variations were observed in the expression of multiple HLA genes among the various groups. In particular, the majority of genes related to HLA exhibited a notably elevated level of expression in groups with low phenotypes when compared to groups with high phenotypes. Furthermore, apart from assessing the expression of HLA genes, we explored the expression of genes associated with immune checkpoint inhibitors and the level of immune cell infiltration in various cohorts of our investigation. Across the groups, we noticed notable variations in the expression of multiple crucial genes related to immune checkpoint. Specifically, the levels of LAG3, CD40, CD276, CD86, HHLA2, TNFRSF8, and other genes varied significantly among the various groups (**Figures 2A, B**). The function of these genes is vital in controlling immune reactions, and their excessive expression may suggest an environment that suppresses the immune system. In addition, our examination uncovered unique trends of immune cell penetration among the categories. The high-phenotype group exhibited notably elevated levels of infiltration by CD8+ T cells and plasma cells in comparison to the low-phenotype group (**Figure 2C**).

### Detection of the genes with altered expression due to apoptosis and development of the genes associated with prognosis in ccRCC population

**Figure 3:**
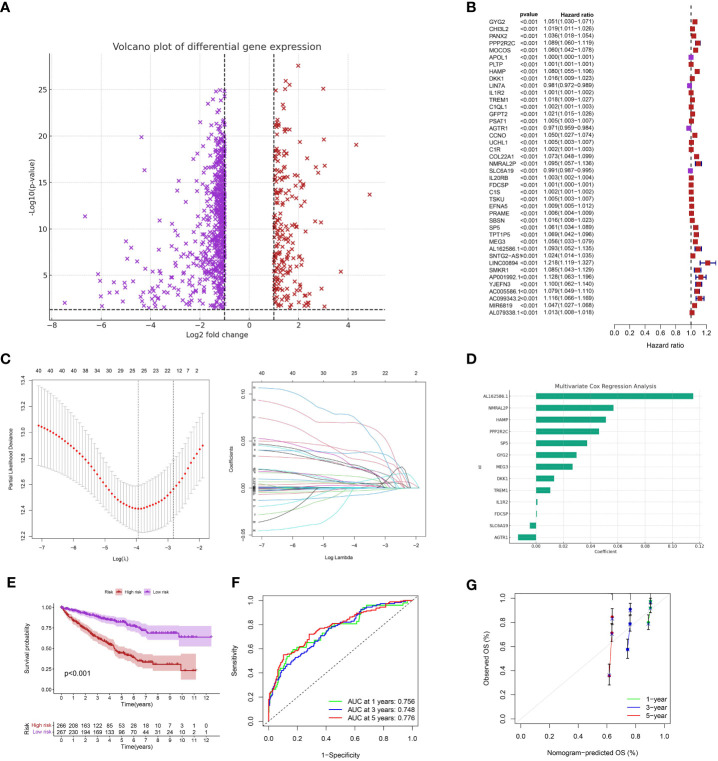
**(A)** The differentially expressed analysis between low- and high-apoptosis groups; **(B)** The univariate COX regression analysis; **(C)** The LASSO regression analysis; **(D)** The multivariate COX regression analysis; **(E)** The survival analysis between low- and high-risk groups; **(F)** The time-dependent ROC curve of the risk model in ccRCC cohort; **(G)** The calibration curve of the risk model.

Initially, we conducted an analysis of differential gene expression and discovered a group of genes that exhibited notable variations in expression levels between the low-apoptosis and high-apoptosis groups (**Figure 3A**). In order to comprehend the predictive capability of these genes with differential expression, we conducted an analysis of univariate Cox regression. Multiple genes were discovered to have a strong correlation with overall survival (OS) (p < 0.001) (**Figure 3B**). To prevent overfitting and identify the most informative genes for prognosis, we utilized LASSO regression analysis on these prognostic genes. We created a predictive model using multivariate Cox regression analysis, utilizing the genes chosen through LASSO regression (**Figure 3C**). The model assigned a risk score to every patient, and then divided them into high-risk and low-risk groups using the median risk score (**Figure 3D**). The risk score was calculated using the formula: Risk score = GYG2 * 0.0297332845768894 + PPP2R2C * 0.0462110263905504 + HAMP * 0.0512445613979477 + DKK1 * 0.0132240157621003 + IL1R2 * 0.00104102088328243 + TREM1 * 0.0104263118963058 + AGTR1 * -0.0131820624746291 + NMRAL2P * 0.0567839789716653 + SLC6A19 * -0.00459902199307787 + FDCSP * 0.00051323964019805 + SP5 * 0.0374983915192158 + MEG3 * 0.0267916578179131 + AL162586.1 * 0.115290461591709. Subsequently, we conducted Kaplan-Meier survival analysis and observed a notable disparity in overall survival (OS) between the high-risk and low-risk cohorts (p < 0.001) (**Figure 3E**). Individuals classified as high-risk experienced a considerably worse outcome in contrast to those classified as low-risk. In addition, we created time-varying receiver operating characteristic (ROC) curves for OS at 1 year, 3 years, and 5 years. Our prognostic model demonstrated excellent predictive accuracy for all time points, as evidenced by the high values of the area under the ROC curve (AUC) (**Figure 3F**). The calibration plots additionally demonstrated a satisfactory concurrence between the projected and observed survival rates at 1-year, 3-year, and 5-year intervals (**Figure 3G**).

### The relationship between the risk score and the infiltration of immune cells and genes related to immune checkpoints

**Figure 4:**
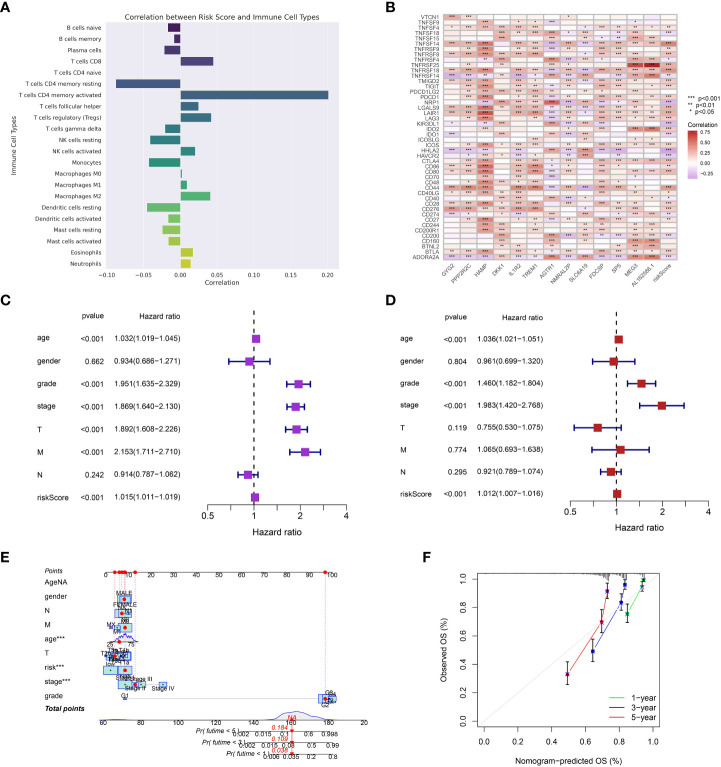
**(A)** The correlation between risk model and immune cell infiltration; **(B)** The correlation between the expression level of immune checkpoint-related genes and risk model; **(C)** The univariate independent prognosis analysis; **(D)** The multivariate independent prognosis analysis; **(E)** The nomogram based on the risk model and clinical features; **(F)** The calibration curve of the nomogram. ***p < 0.001.

Initially, we examined the correlation among risk scores, infiltration of immune cells, and the expression of genes associated with immune checkpoint inhibitors. A strong positive relationship was discovered between risk scores and the extent of infiltration of various types of immune cells (**Figure 4A**). This implies that individuals with elevated risk scores might possess a unique immune environment. Moreover, the risk scores showed a significant correlation with the expression of various immune checkpoint genes, such as TNFSF4, TNFSF18, TNFSF14, TNFRSF9, TNFRSF8, TNFRSF4, TNFRSF25, and others (**Figure 4B**).

### The relationship between the risk score and clinical characteristics

Afterwards, we investigated the correlation between risk scores and different clinical attributes. The overall survival was significantly associated with various clinical characteristics, such as age, grade, stage, T stage, M stage, and risk score, as indicated by the results of the univariate Cox regression analysis (**Figure 4C**). After accounting for other clinical characteristics in the multivariate Cox regression analysis, the risk score persisted as a separate prognostic indicator for overall survival (**Figure 4D**). Next, we developed a nomogram that combines the risk score and important clinical characteristics to offer a quantitative approach for estimating a patient’s likelihood of surviving for 1 year, 3 years, and 5 years. According to the nomogram, prognosis was primarily influenced by the risk score, with tumor stage and age also playing significant roles (**Figure 4E**). The calibration graphs for the likelihood of surviving for 1 year, 3 years, and 5 years demonstrated a favorable concurrence between the forecast made by the nomogram and the real-life observation (**Figure 4F**).

### Somatic mutations in ccRCC cohort

**Figure 5:**
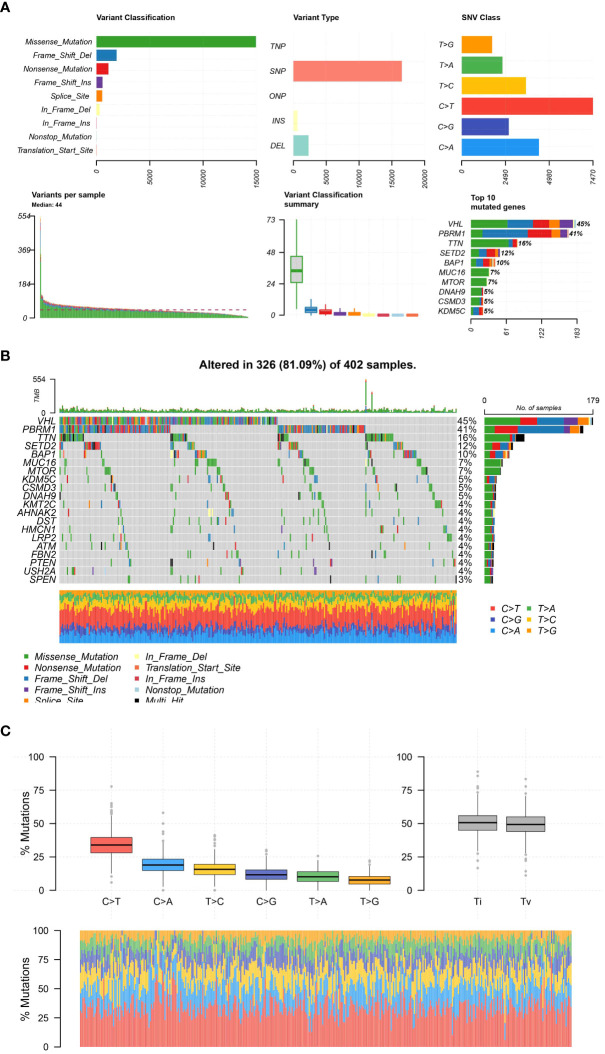
**(A)**; The plot representing the distribution and types of mutations; **(B)** The figure shows a waterfall plot illustrating the mutation landscape of the analyzed tumor samples. Each column represents an individual sample, and each colored cell within the column denotes the presence of a particular gene mutation. The color coding is explained in the inset key. The top bar plot shows the mutation burden per sample, and the right-hand bar plot displays the frequency of mutations for each gene across all samples; **(C)** The different mutation types of all samples.

During our examination of the TCGA Kidney Renal Clear Cell Carcinoma (KIRC) dataset, we investigated the spectrum of genetic alterations in patients with KIRC. The examination uncovered a significant number of mutations in the KIRC specimens. VHL, the gene with the highest mutation frequency, was found to be mutated in 45% of the KIRC samples. Furthermore, the KIRC cohort exhibits a high mutation rate in genes such as VHL, PBRM1, TTN, SETD2, BAP1, MUC16, MTOR, DNAH9, CSMD3, and KDM5C, which are among the top 10 mutated genes. Furthermore, we also assess the association between the mutation category and the numerous genes in the ccRCC population. Moreover, the majority of the mutations were primarily of the Missense_Mutation category. Taken together, our findings provide a comprehensive view of the somatic mutation landscape in KIRC. Identifying commonly and significantly mutated genes may enhance comprehension of the molecular mechanisms of KIRC and potentially direct the advancement of more precise therapeutic approaches (**Figures 5A-C**).

### The relationship between clinical characteristics and risk assessment

**Figure 6:**
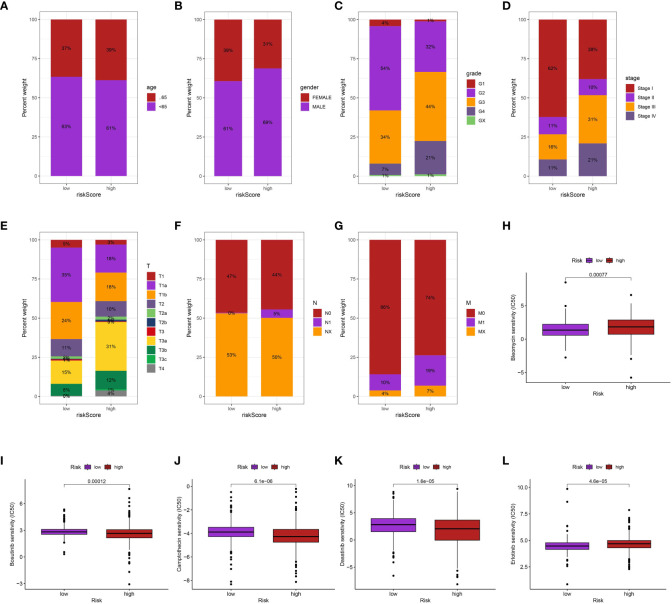
The correlation between risk score and age **(A)**, gender **(B)**, grade **(C)**, stage **(D)**, T stage **(E)**, N stage **(F)**, M stage **(G)**; The drug sensitive analysis of Bleomycin **(H)**, Bosutinib **(I)**, Camptothecin **(J)**, Dasatinib **(K)** and Erlotinib **(L)** between low- and high-risk groups.

Furthermore, we investigate the association between clinical characteristics and risk score. The findings indicate a positive correlation between elevated risk score and increased grade, stage, T stage, and M stage. Furthermore, the male ccRCC individuals were also associated with the elevated risk score (**Figures 6A–G**).

### Analyzing the correlation between risk scores and drug sensitivity in the ccRCC cohort

Additionally, we performed an investigation to examine the correlation between the risk scores of ccRCC individuals, obtained from our prognostic algorithm, and drug responsiveness utilizing information from The Cancer Genome Atlas (TCGA). The correlation between the sensitivity of various drugs and the risk scores was found to be significant. In particular, individuals with elevated risk scores exhibited greater susceptibility to bleomycin and erlotinib in contrast to those with lower risk scores (p < 0.05). On the other hand, patients with high-risk scores may not have a favorable response to bosutinib, camptothecin, and dasatinib (p < 0.05), indicating a correlation between highrisk scores and treatment resistance. The utilization of risk scores to inform treatment decisions offers a fresh perspective and has the potential to aid in the creation of individualized therapeutic approaches for patients with ccRCC *(* **Figures 6H–L**).

### The overexpression of the DKK1 promote the cell apoptosis in RCC cells

**Figure 7:**
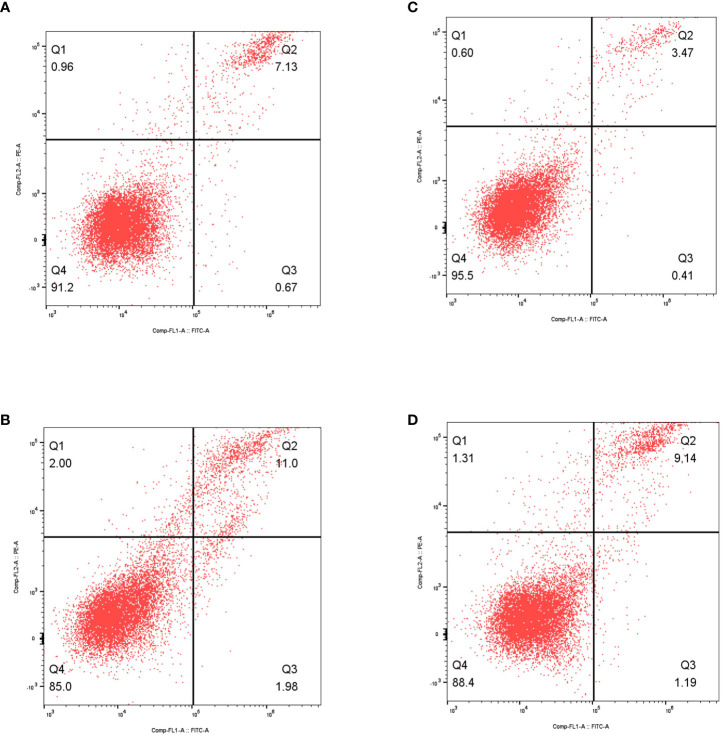
**(A)** The results of flow cytometry revealed the apoptosis in non-treated 786-O cells; **(B)** The results of flow cytometry revealed the apoptosis in over-expressed DKK1 786-O cells; **(C)** The results of flow cytometry revealed the apoptosis in non-treated Caki-1 cells; **(D)** The results of flow cytometry revealed the apoptosis in over-expressed DKK1 Caki-1 cells.

Additionally, to investigate the connection between DKK1 and programmed cell death, we conducted flow cytometry analysis to assess cell apoptosis. The findings indicated that the increased expression of DKK1 enhanced the capacity of cell apoptosis in both 786-O and Caki-1 cell lines (**Figure 7**).

## Discussion

Our research has provided valuable knowledge about the various characteristics associated with clear cell renal cell carcinoma (ccRCC) and has made significant progress in identifying potential markers for predicting the prognosis and treatment of this condition. Categorizing ccRCC patients into low- and high-phenotype groups using the ssGSEA scores has offered a unique viewpoint on the clinical landscape of the disease. For localized ccRCC, surgical intervention, including partial or radical nephrectomy, is typically the first-line treatment (15). The treatment options for advanced or metastatic ccRCC have greatly improved due to the introduction of targeted therapies, such as sunitinib and pazopanib, which are tyrosine kinase inhibitors (TKIs), as well as immune checkpoint inhibitors like nivolumab and ipilimumab (16).

The notable variations observed in the manifestation of Human Leukocyte Antigen (HLA) genes and crucial immune checkpoint genes among these phenotype groups emphasize the intricate interaction between ccRCC and the immune system. The results mirror the well-known impact of the immune environment on the advancement of tumors, the effectiveness of treatment, and the survival of patients. Specifically, our examination of increased amounts of CD8+ T lymphocyte and plasma cell invasion in high-phenotype categories suggests a possible tactic of immune evasion utilized by aggressive ccRCC subtypes.

The performance of our predictive model, developed using genes that show differential expression in relation to apoptosis, has proven to be strong in categorizing the risk of ccRCC patients. The notable disparities in survival rates between the high-risk and low-risk categories, along with the model’s impressive ability to predict accurately, emphasize the potential of incorporating molecular information into clinical prognosis. Significantly, the effectiveness of the model was further strengthened by its classification as a standalone prognostic factor in the multivariate Cox regression analysis. Over the past few years, numerous research studies have concentrated on examining the significance of risk models in the treatment of cancer. In a recent study, it was found that the gene GLS2 is upregulated in ccRCC cells during ferroptosis, according to researchers’ findings. Moreover, ccRCC cells that were subjected to GLS2 shRNA treatment displayed lower rates of survival, diminished levels of glutathione, and increased levels of lipid peroxide. The results indicate that GLS2 might function as an inhibitor of ferroptosis in ccRCC (17).

Our findings indicate that the connections we formed among risk scores, infiltration of immune cells, expression of genes related to immune checkpoint inhibitors, and different clinical characteristics propose that our scoring system for risk could function as a comprehensive gauge of the severity of the disease. This enhances the possibility of our risk assessment as a valuable instrument for categorizing patients and providing treatment recommendations. Additional context is provided to our findings through our analysis of the mutational landscape in the TCGA Kidney Renal Clear Cell Carcinoma (KIRC) dataset. Understanding the molecular mechanisms underlying ccRCC is enhanced by the occurrence of missense mutations and the discovery of commonly mutated genes like VHL and PBRM1.These findings also raise the possibility of developing targeted therapeutic strategies based on specific genetic alterations. The connection between risk scores and drug sensitivity is especially fascinating, indicating the possibility of our risk scores in guiding decisions on treatment and contributing to individualized therapeutic approaches. Nevertheless, additional investigation is required to confirm these connections and to examine their practical significance.

The atypical manifestation of Dickkopf-1 (Dkk1) is recognized to play a role in the progression of different forms of malignancies. Dkk1 is a member of a protein group comprising Dkk2, Dkk3, and Dkk4. All these proteins are secreted and possess comparable conserved cysteine domains. The Dkk family primarily operates by obstructing the Wnt/b-catenin pathway, leading to the breakdown of B-catenin through the proteasome, the initiation of apoptosis, and the cessation of cellular proliferation (18). During the course of this study, we made a significant finding indicating that DKK1 might have a crucial involvement in the induction of apoptosis in individuals with ccRCC. In the ccRCC cohort, the excessive expression of DKK1 may enhance the capacity for apoptosis.

Our study, while providing novel insights, has its limitations. To validate our findings, it is important to conduct prospective studies due to the biases present in publicly available datasets and the retrospective nature of this study. In spite of these difficulties, our research signifies a significant advancement in comprehending the intricate biology of ccRCC and offers hope for enhancing patient prognosis and tailoring treatment methods.

## Data availability statement

The original contributions presented in the study are included in the article/**Supplementary Material**. Further inquiries can be directed to the corresponding authors.

## Author contributions

XZhai: Conceptualization, Writing – review & editing. XC: Investigation, Writing – original draft. JG: Software, Writing – original draft. DG: Investigation, Writing – original draft. XZhan: Software, Writing – original draft. MT: Conceptualization, Writing – review & editing. DX: Software, Writing – original draft.

## References

[1] Hsieh JJ Purdue MP Signoretti S Swanton C Albiges L Schmidinger M. Renal cell carcinoma. Nat Rev Dis Primers. (2017) 3:17009. doi: 10.1038/nrdp.2017.9 28276433 PMC5936048

[2] Zhao J Eyzaguirre E. Clear cell papillary renal cell carcinoma. Arch Pathol Lab Med (2019) 143(9):1154–8. doi: 10.5858/arpa.2018-0121-RS 30672334

[3] Garje R Elhag D Yasin HA Acharya L Vaena D Dahmoush L. Comprehensive review of chromophobe renal cell carcinoma. Crit Rev Oncol Hematol (2021) 160:103287. doi: 10.1016/j.critrevonc.2021.103287 33753250

[4] Makhov P Joshi S Ghatalia P Kutikov A Uzzo RG Kolenko VM. Resistance to systemic therapies in clear cell renal cell carcinoma: mechanisms and management strategies. Mol Cancer Ther (2018) 17(7):1355–64. doi: 10.1158/1535-7163.MCT-17-1299 PMC6034114 29967214

[5] Linehan WM Ricketts CJ. The Cancer Genome Atlas of renal cell carcinoma: findings and clinical implications. Nat Rev Urol. (2019) 16(9):539–52. doi: 10.1038/s41585-019-0211-5 31278395

[6] Bui TO Dao VT Nguyen VT Feugeas JP Pamoukdjian F Bousquet G. Genomics of clear-cell renal cell carcinoma: a systematic review and meta-analysis. Eur Urol. (2022) 81(4):349–61. doi: 10.1016/j.eururo.2021.12.010 34991918

[7] Díaz-Montero CM Rini BI Finke JH. The immunology of renal cell carcinoma. Nat Rev Nephrol. (2020) 16(12):721–35. doi: 10.1038/s41581-020-0316-3 32733094

[8] Williamson SR Gill AJ Argani P Chen YB Egevad L Kristiansen G. Report from the international society of urological pathology (ISUP) consultation conference on molecular pathology of urogenital cancers: III: molecular pathology of kidney cancer. Am J Surg Pathol (2020) 44(7):e47–65. doi: 10.1097/PAS.0000000000001476 PMC7289677 32251007

[9] Ahrens M Scheich S Hartmann A Bergmann L. IAG-N interdisciplinary working group kidney cancer of the german cancer society. Non-clear cell renal cell carcinoma - pathology and treatment options. Oncol Res Treat (2019) 42(3):128–35. doi: 10.1159/000495366 30799404

[10] Kim IH Lee HJ. The frontline immunotherapy-based treatment of advanced clear cell renal cell carcinoma: current evidence and clinical perspective. Biomedicines. (2022) 10(2):251. doi: 10.3390/biomedicines10020251 35203461 PMC8869224

[11] Kasherman L Siu DHW Woodford R Harris CA. Angiogenesis inhibitors and immunomodulation in renal cell cancers: the past, present, and future. Cancers (Basel). (2022) 14(6):1406. doi: 10.3390/cancers14061406 35326557 PMC8946206

[12] Doppalapudi SK Leopold ZR Thaper A Kaldany A Chua K Patel HV. Clearing up clear cell: clarifying the immuno-oncology treatment landscape for metastatic clear cell RCC. Cancers (Basel). (2021) 13(16):4140. doi: 10.3390/cancers13164140 34439293 PMC8391664

[13] Climent C Soriano S Bonfill T Lopez N Rodriguez M Sierra M. The role of immunotherapy in non-clear cell renal cell carcinoma. Front Oncol (2023) 13:941835. doi: 10.3389/fonc.2023.941835 36816976 PMC9936973

[14] Patel HV Srivastava A Srinivasan R Singer EA. A challenging frontier - the genomics and therapeutics of nonclear cell renal cell carcinoma. Curr Opin Oncol (2021) 33(3):212–20. doi: 10.1097/CCO.0000000000000721 PMC8244822 33818540

[15] George DJ Lee CH Heng D. New approaches to first-line treatment of advanced renal cell carcinoma. Ther Adv Med Oncol (2021) 11:13:17588359211034708. doi: 10.1177/17588359211034708 PMC8435931 34527080

[16] Zarrabi K Walzer E Zibelman M. Immune checkpoint inhibition in advanced non-clear cell renal cell carcinoma: leveraging success from clear cell histology into new opportunities. Cancers (Basel). (2021) 13(15):3652. doi: 10.3390/cancers13153652 34359554 PMC8344970

[17] Shi Z Zheng J Liang Q Liu Y Yang Y Wang R. Identification and validation of a novel ferroptotic prognostic genes-based signature of clear cell renal cell carcinoma. Cancers (Basel). (2022) 14(19):4690. doi: 10.3390/cancers14194690 36230613 PMC9562262

[18] Zhu G Song J Chen W Yuan D Wang W Chen X. Expression and role of dickkopf-1 (Dkk1) in tumors: from the cells to the patients. Cancer Manag Res (2021) 13:659–75. doi: 10.2147/CMAR.S275172 PMC7847771 33536782

