# Supplementary figures and images for: The stratification and prognostic importance of molecular and immune landscapes in clear cell renal cell carcinoma
Source: Front Oncol. 2023 Oct 2;13:1256720. doi: 10.3389/fonc.2023.1256720 (PMC10577421; doi:10.3389/fonc.2023.1256720)

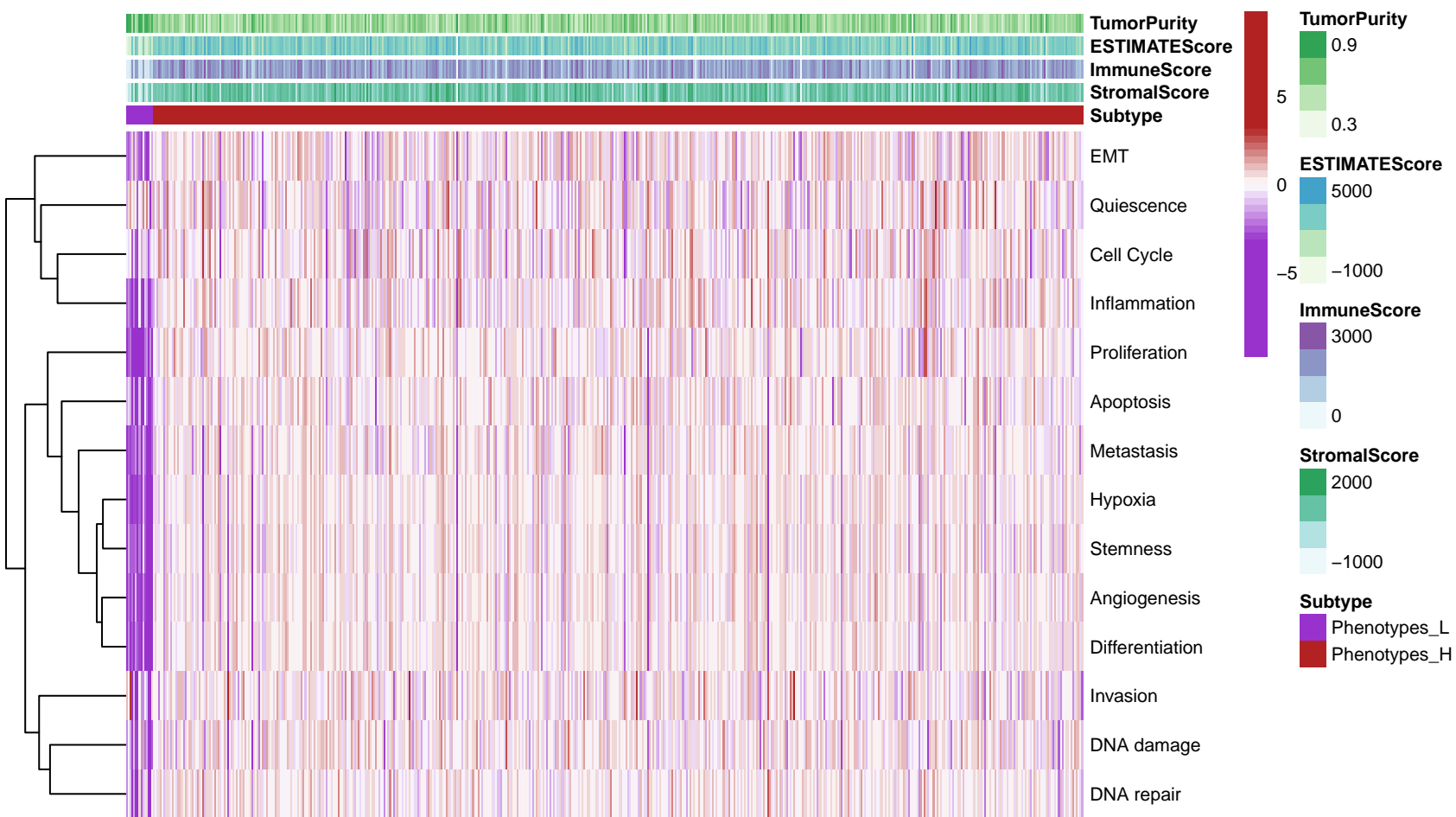

Supplement: Supplementary file 1 [file DataSheet_1.zip › estimateHM.pdf]

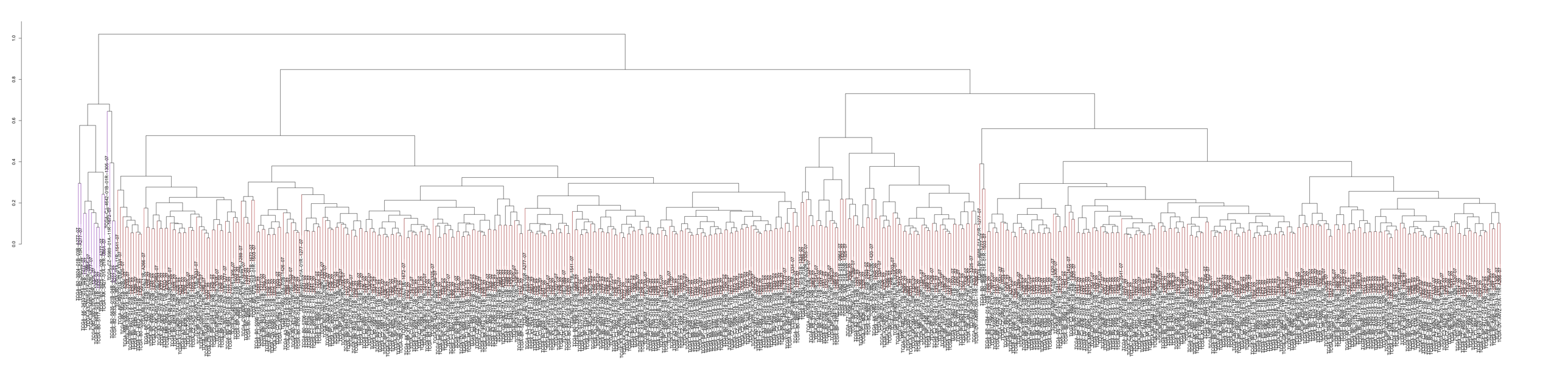

Supplement: Supplementary file 1 [file DataSheet_1.zip › hclust.pdf]

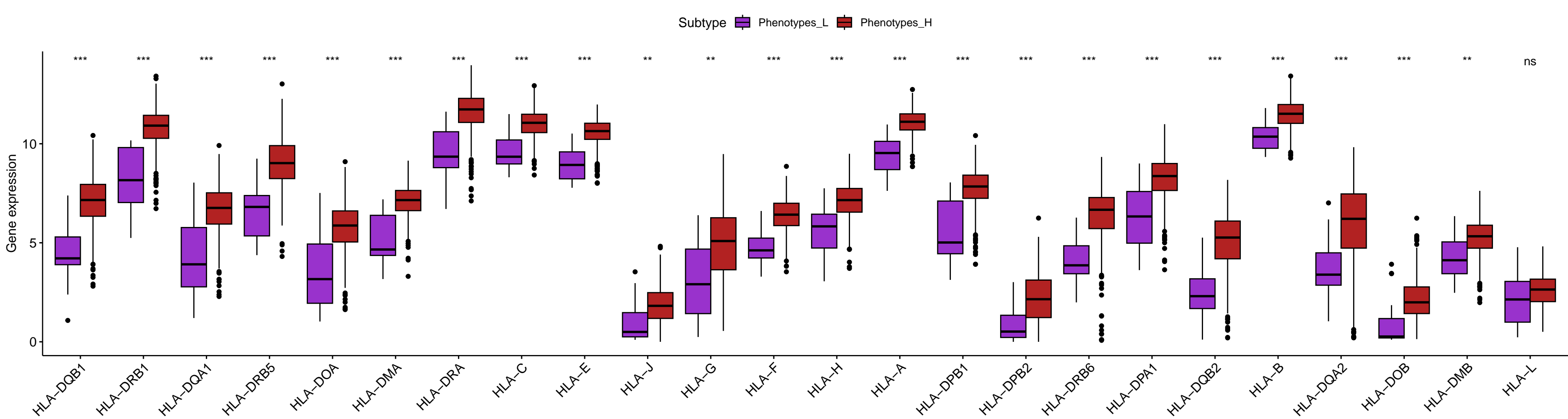

Supplement: Supplementary file 1 [file DataSheet_1.zip › HLA.pdf]

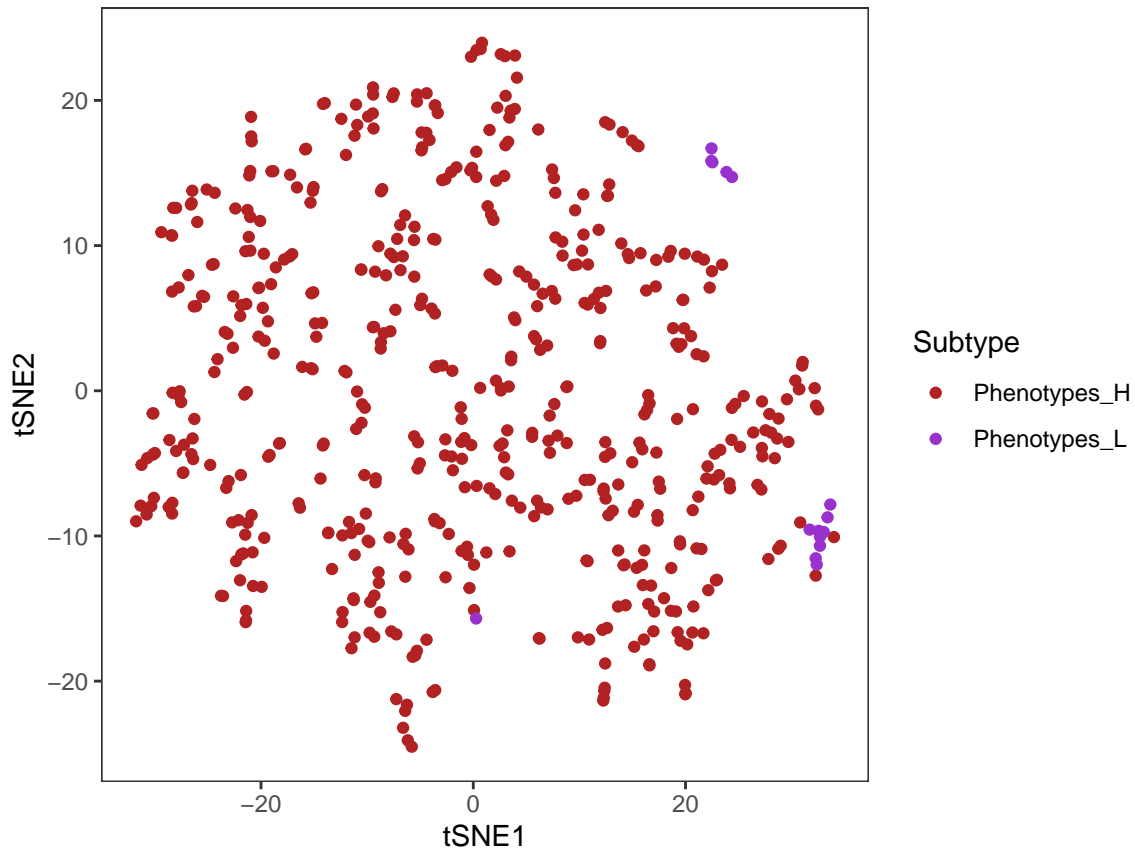

Supplement: Supplementary file 1 [file DataSheet_1.zip › tSNE.pdf]

Subtype Phenotypes\_L Phenotypes\_H

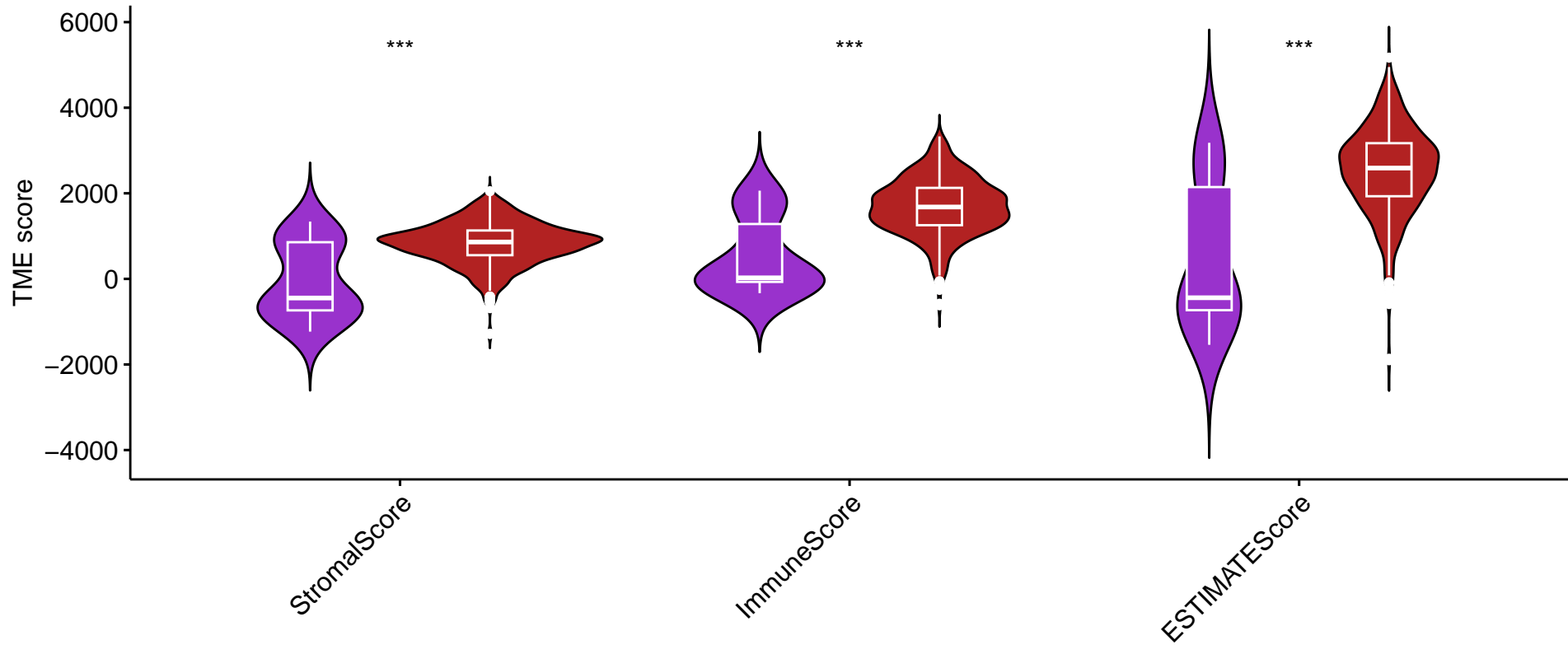

Supplement: Supplementary file 1 [file DataSheet_1.zip › vioplot.pdf]

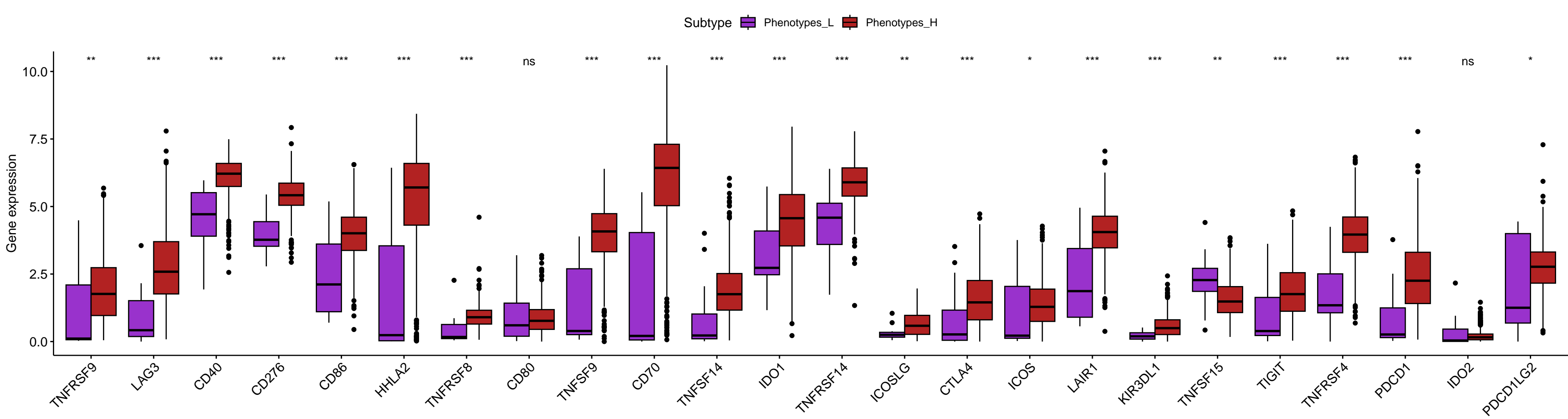

Supplement: Supplementary file 2 [file DataSheet_2.zip › cp1.pdf]

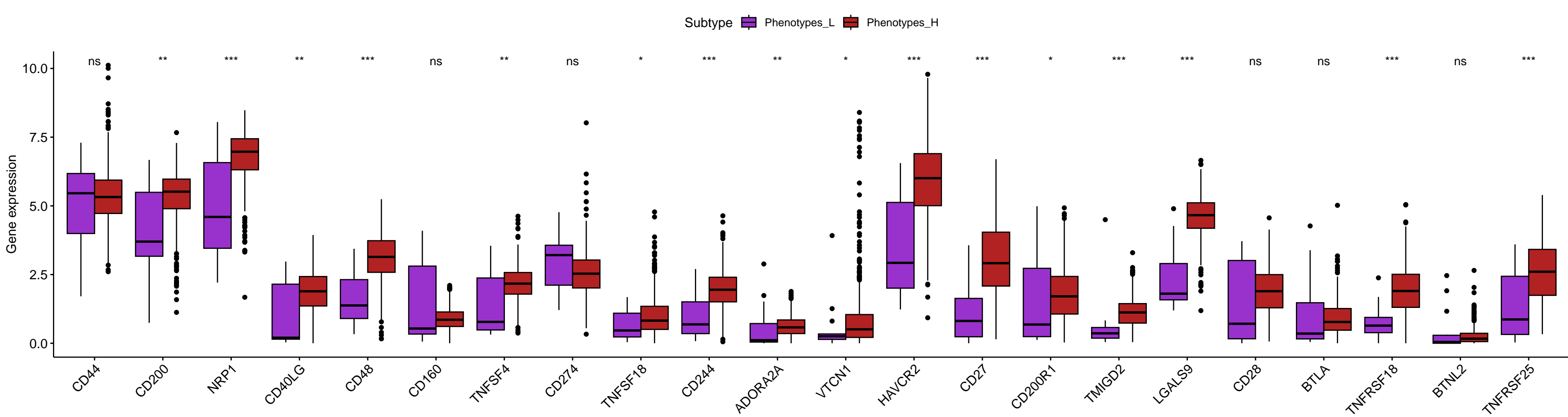

Supplement: Supplementary file 2 [file DataSheet_2.zip › cp2.pdf]

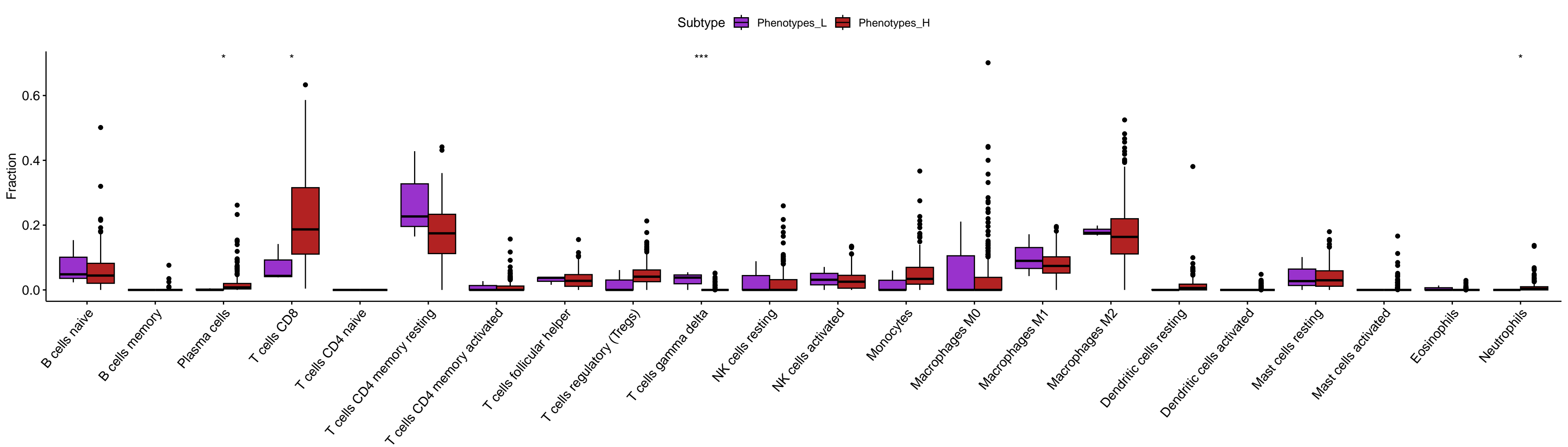

Supplement: Supplementary file 2 [file DataSheet_2.zip › immune.diff.pdf]

Partial Likelihood Deviance

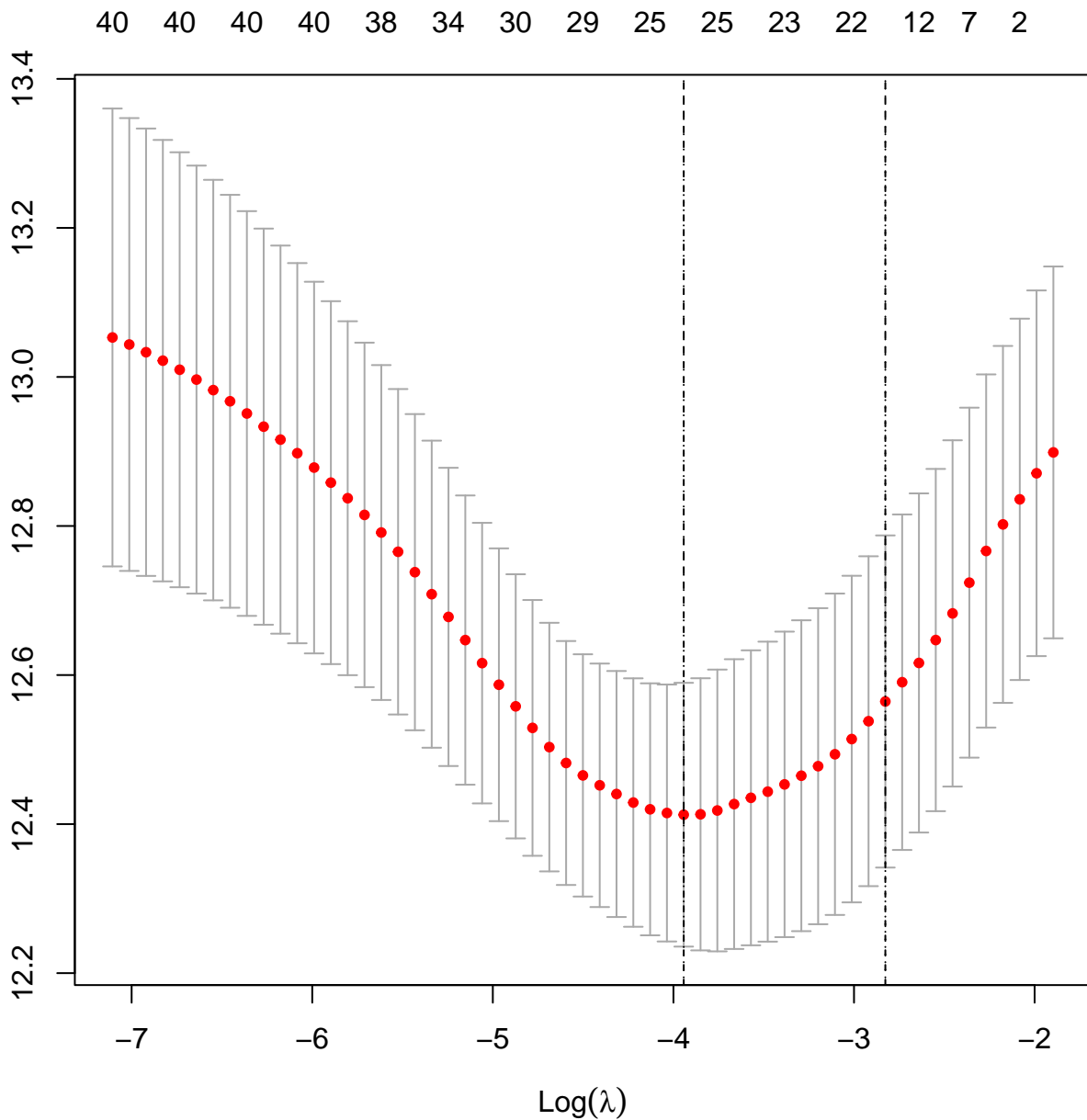

Supplement: Supplementary file 3 [file DataSheet_3.zip › lasso.cvfit.pdf]

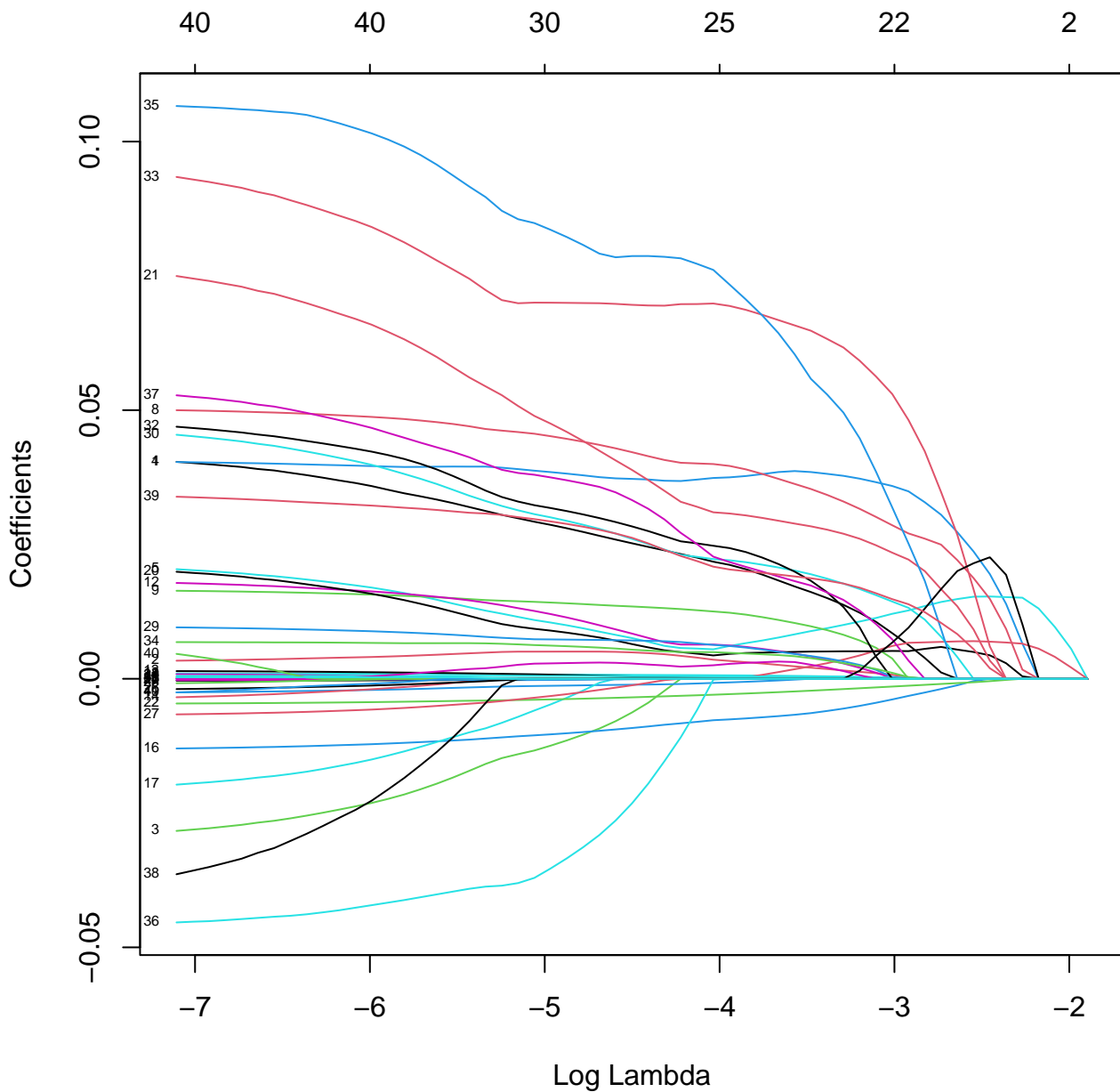

Supplement: Supplementary file 3 [file DataSheet_3.zip › lasso.lambda.pdf]

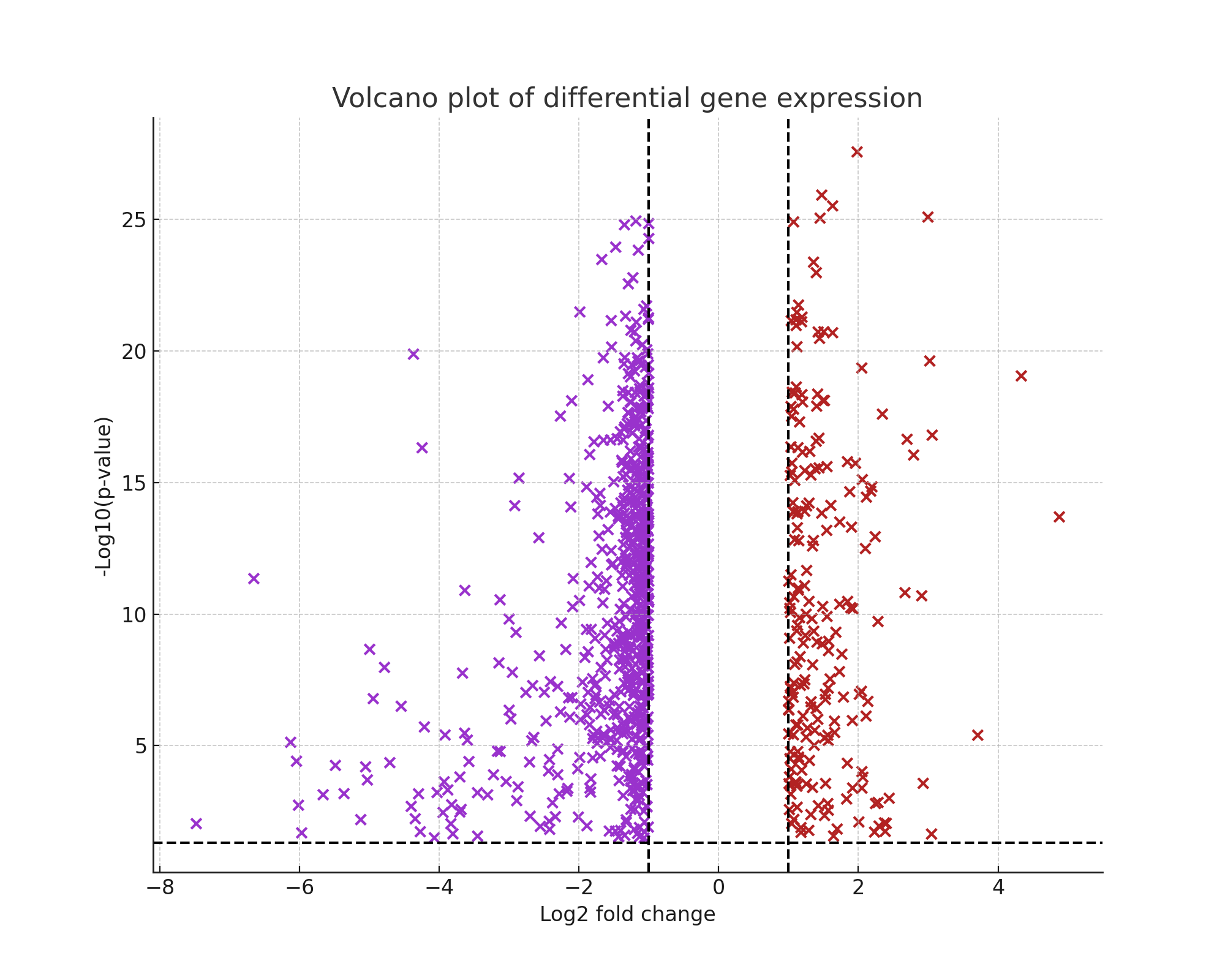

Supplement: Supplementary file 3 [file DataSheet_3.zip › volcano_plot.png]

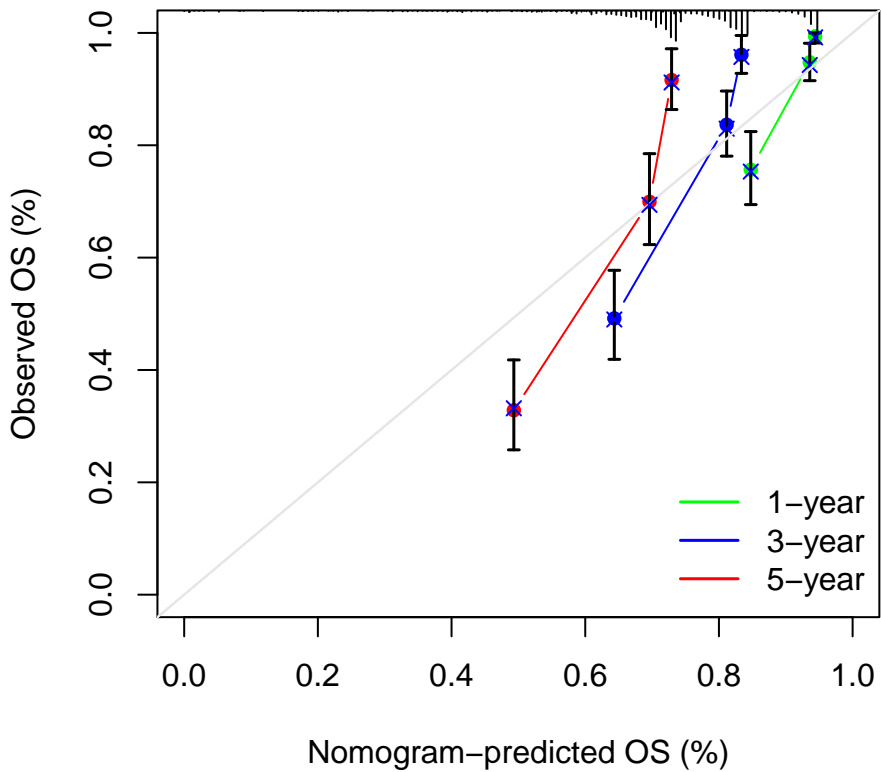

Supplement: Supplementary file 4 [file DataSheet_4.zip › calibration.pdf]

|           | pvalue | Hazard ratio       |
|-----------|--------|--------------------|
| age       | <0.001 | 1.036(1.021–1.051) |
| gender    | 0.804  | 0.961(0.699–1.320) |
| grade     | <0.001 | 1.460(1.182–1.804) |
| stage     | <0.001 | 1.983(1.420–2.768) |
| T         | 0.119  | 0.755(0.530–1.075) |
| M         | 0.774  | 1.065(0.693–1.638) |
| N         | 0.295  | 0.921(0.789–1.074) |
| riskScore | <0.001 | 1.012(1.007–1.016) |

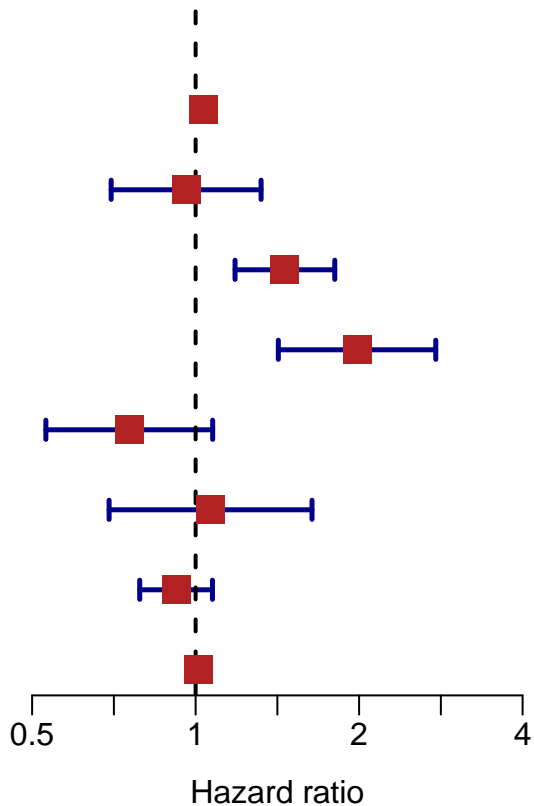

Supplement: Supplementary file 4 [file DataSheet_4.zip › multiForest.pdf]

Points

AgeNA

gender

N

M

age\*\*\*

T

risk\*\*\*

stage\*\*\*

grade

**Total points**

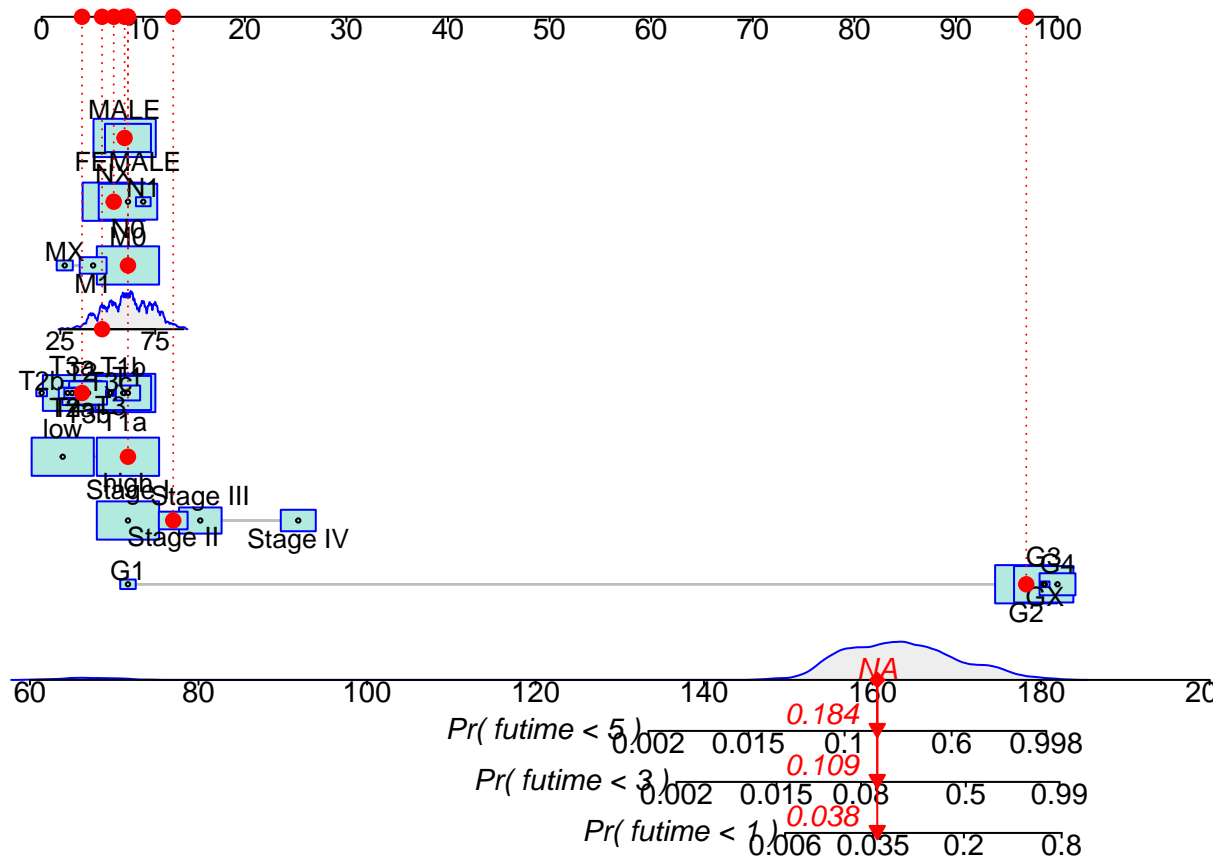

Supplement: Supplementary file 4 [file DataSheet_4.zip › Rplot03.pdf]

|           | pvalue | Hazard ratio       |
|-----------|--------|--------------------|
| age       | <0.001 | 1.032(1.019–1.045) |
| gender    | 0.662  | 0.934(0.686–1.271) |
| grade     | <0.001 | 1.951(1.635–2.329) |
| stage     | <0.001 | 1.869(1.640–2.130) |
| T         | <0.001 | 1.892(1.608–2.226) |
| M         | <0.001 | 2.153(1.711–2.710) |
| N         | 0.242  | 0.914(0.787–1.062) |
| riskScore | <0.001 | 1.015(1.011–1.019) |

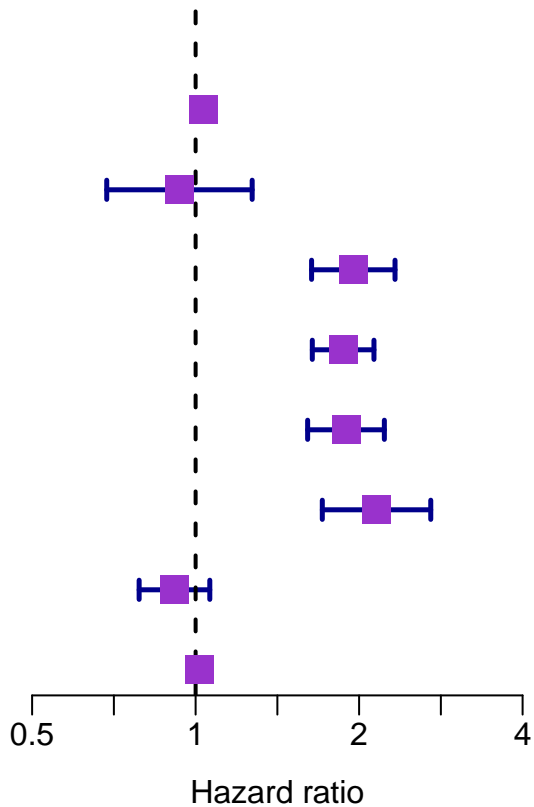

Supplement: Supplementary file 4 [file DataSheet_4.zip › uniForest.pdf]

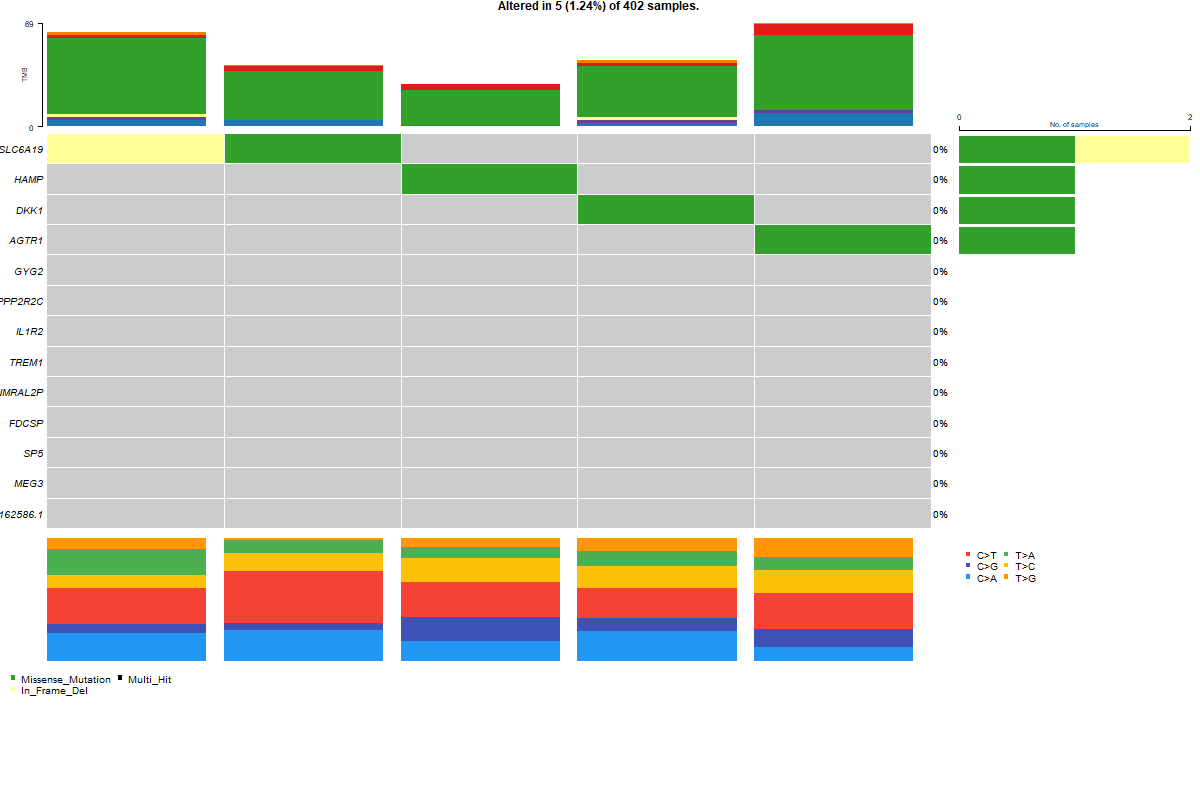

Supplement: Supplementary file 5 [file DataSheet_5.zip › oncoplot.png]

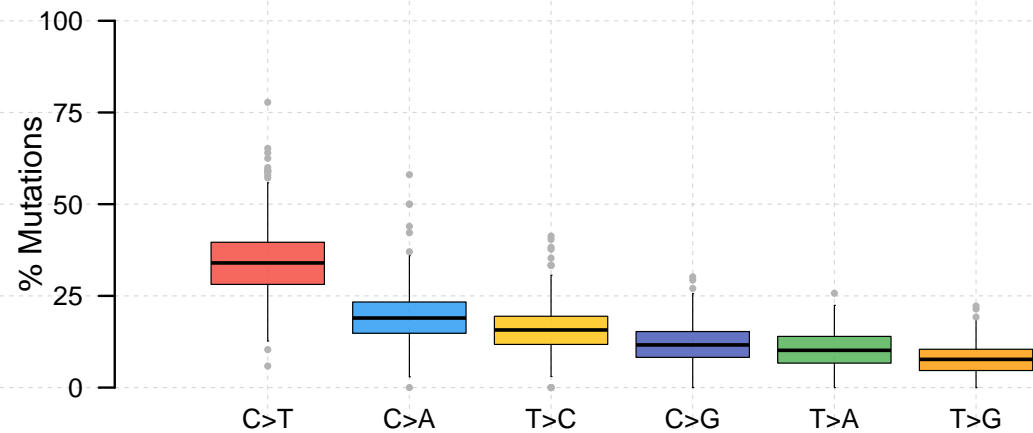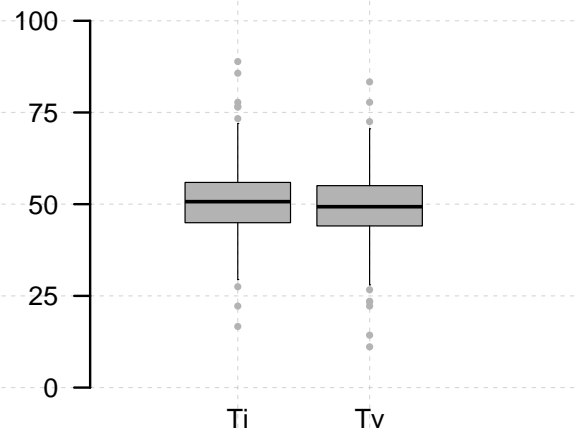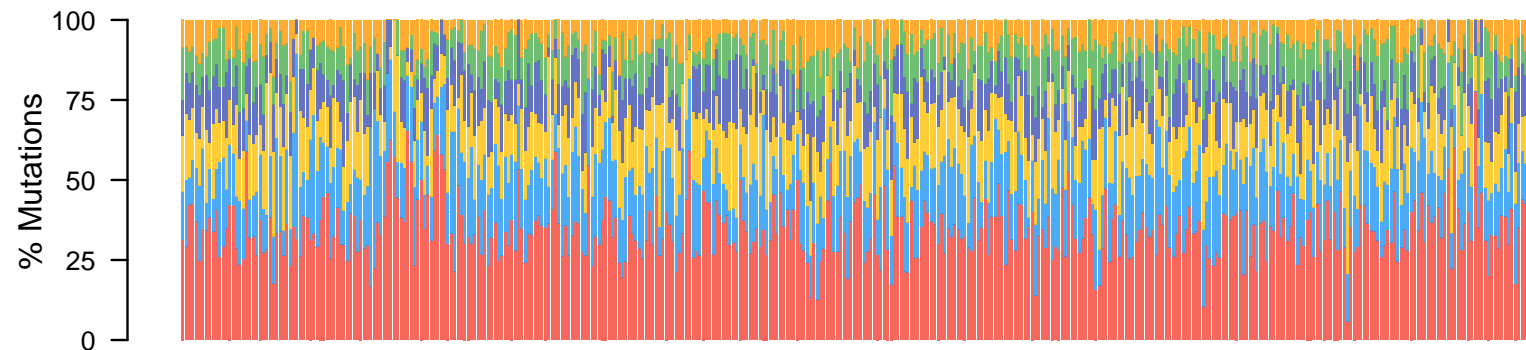

Supplement: Supplementary file 5 [file DataSheet_5.zip › Rplot02.pdf]

Altered in 5 (1.24%) of 402 samples.

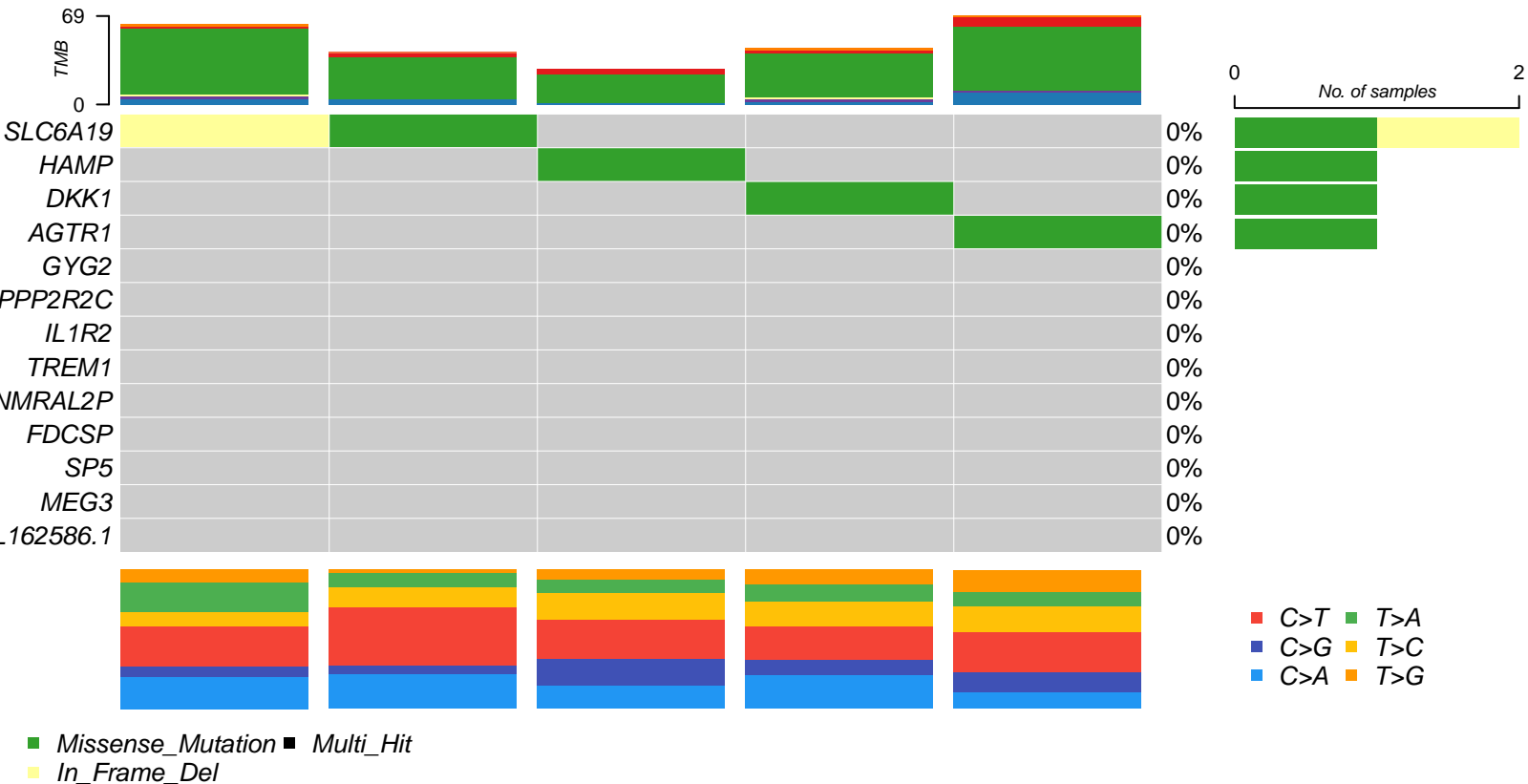

Supplement: Supplementary file 5 [file DataSheet_5.zip › Rplot04.pdf]

Percent weight

100  
75  
50  
25  
0

low

high

riskScore

37%

39%

63%

61%

age

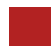

..65

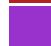

<65

Supplement: Supplementary file 6 [file DataSheet_6.zip › age.barplot.pdf]

Percent weight

100  
75  
50  
25  
0

low

high

riskScore

gender

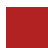

FEMALE

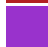

MALE

39%

31%

61%

69%

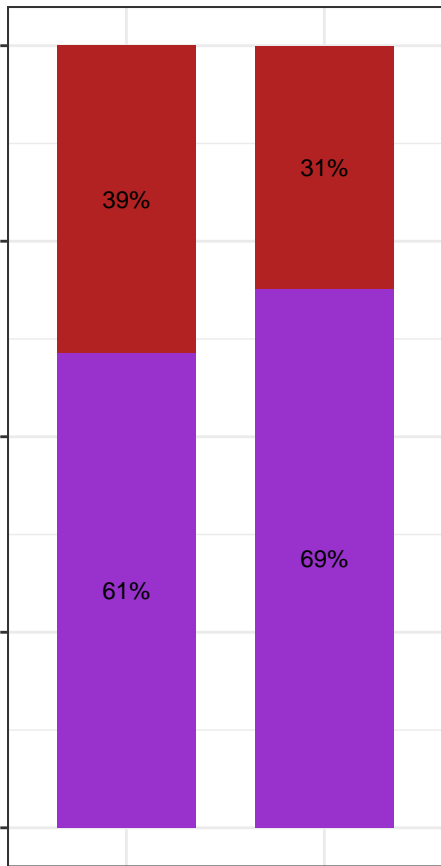

Supplement: Supplementary file 6 [file DataSheet_6.zip › gender.barplot.pdf]

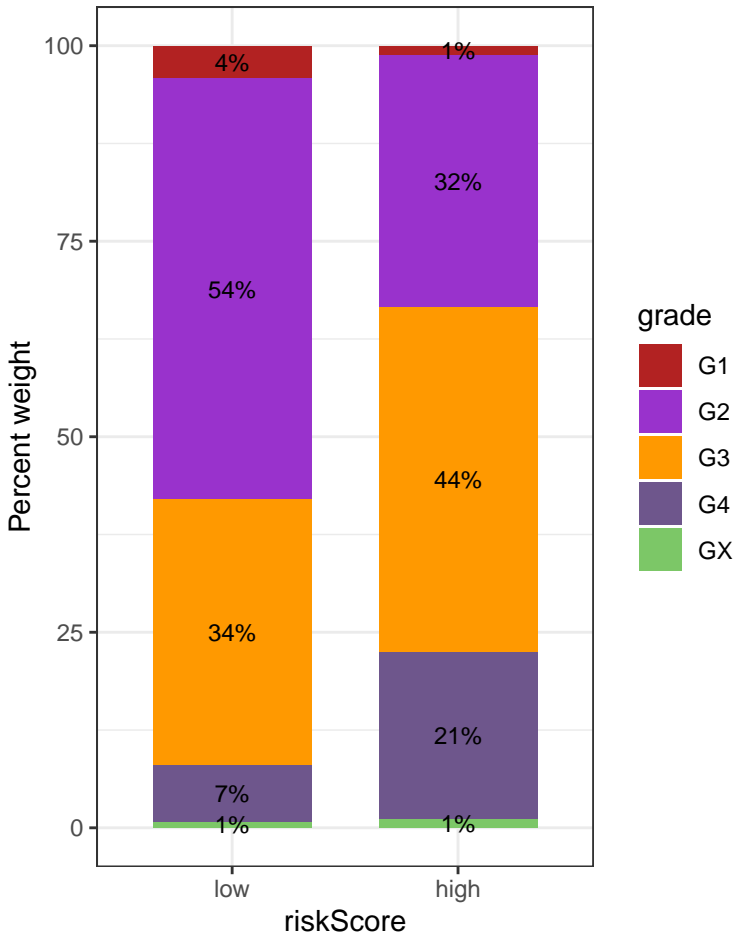

Supplement: Supplementary file 6 [file DataSheet_6.zip › grade.barplot.pdf]

Percent weight

100  
75  
50  
25  
0

low

high

riskScore

86%

10%

4%

74%

19%

7%

M

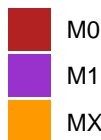

Supplement: Supplementary file 6 [file DataSheet_6.zip › M.barplot.pdf]

Percent weight

100  
75  
50  
25  
0

low

high

riskScore

47%

44%

0%

5%

53%

50%

N

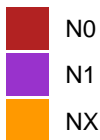

N0

N1

NX

Supplement: Supplementary file 6 [file DataSheet_6.zip › N.barplot.pdf]

Percent weight

100  
75  
50  
25  
0

low

high

riskScore

stage

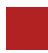

Stage I

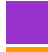

Stage II

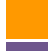

Stage III

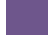

Stage IV

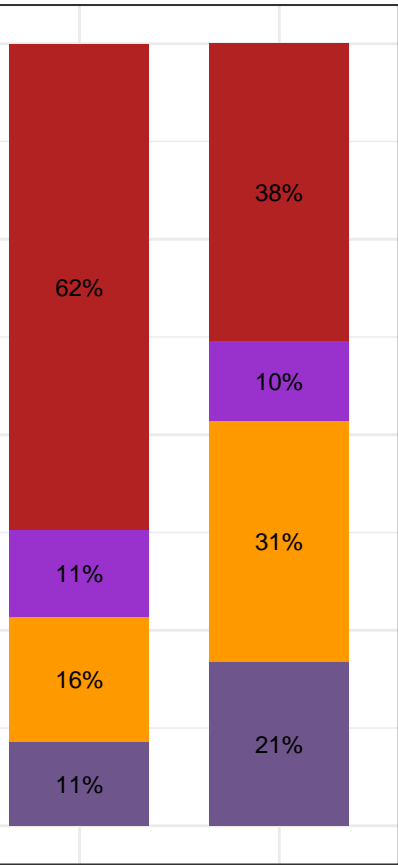

Supplement: Supplementary file 6 [file DataSheet_6.zip › stage.barplot.pdf]

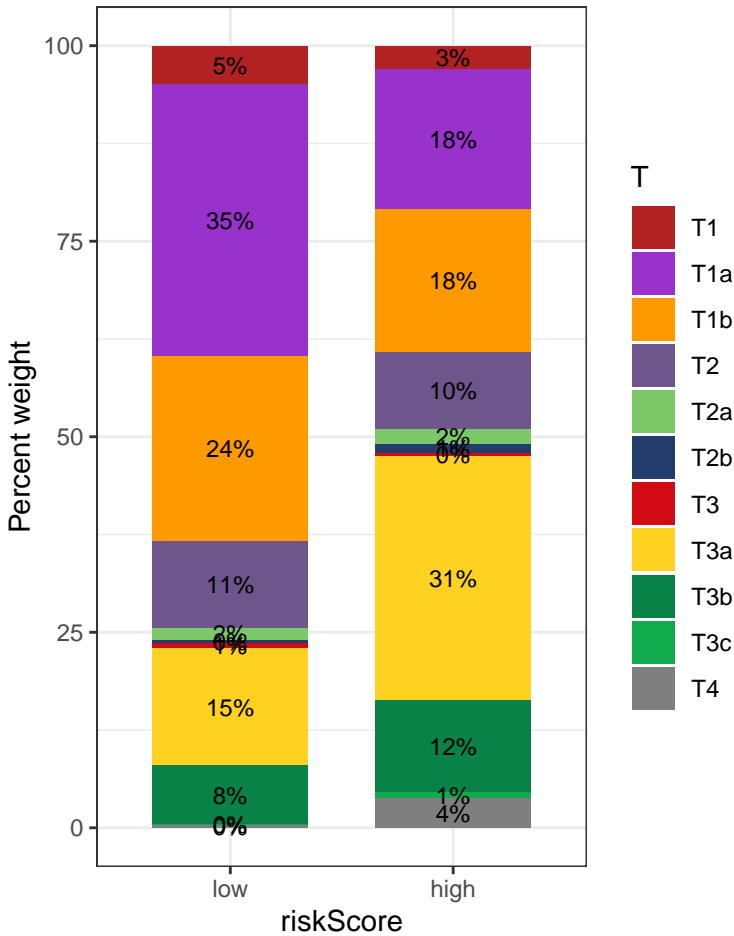

Supplement: Supplementary file 6 [file DataSheet_6.zip › T.barplot.pdf]

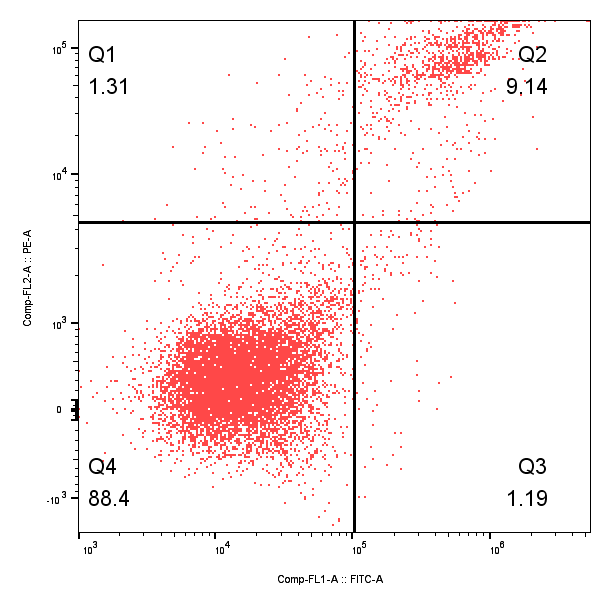

Supplement: Supplementary file 7 [file DataSheet_7.zip › 1.png]

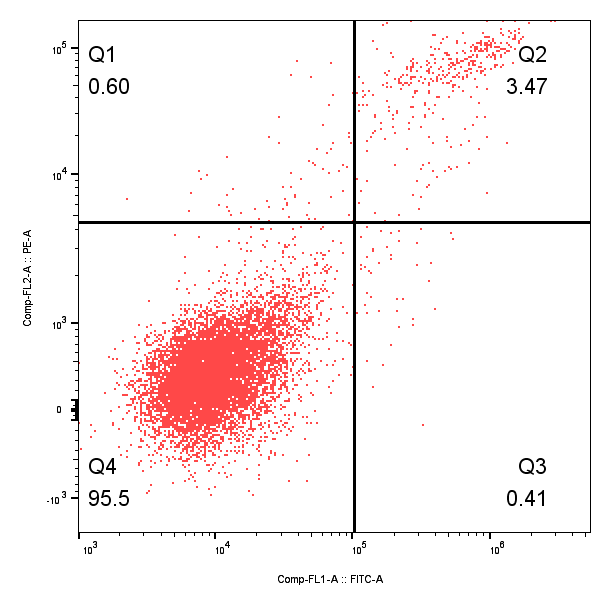

Supplement: Supplementary file 7 [file DataSheet_7.zip › 2.png]

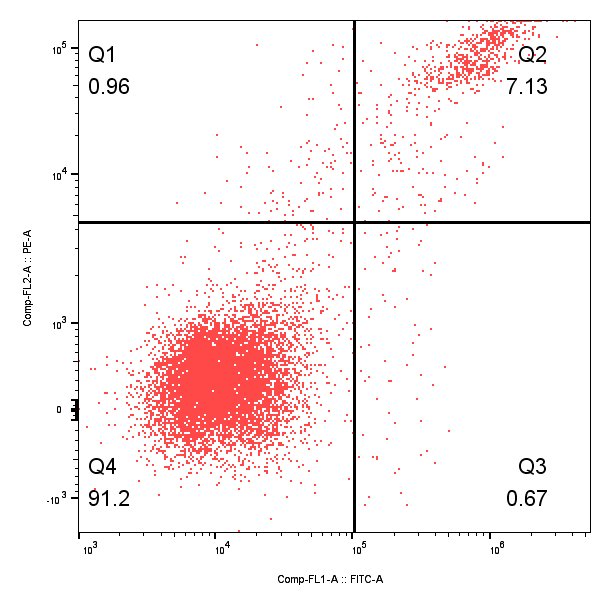

Supplement: Supplementary file 7 [file DataSheet_7.zip › 3.png]

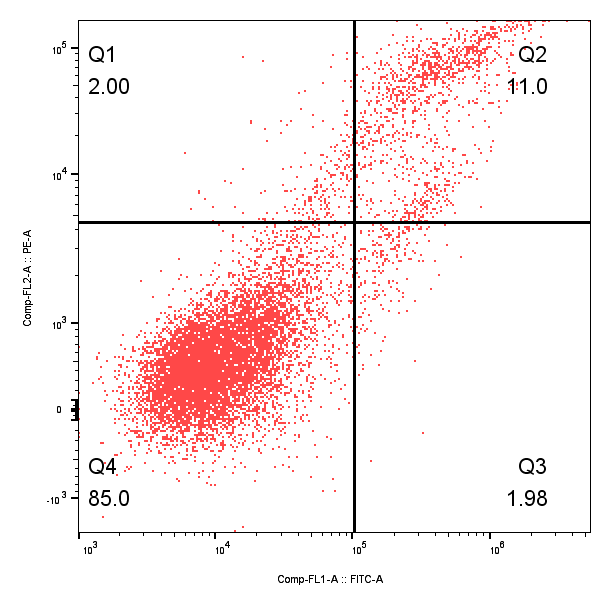

Supplement: Supplementary file 7 [file DataSheet_7.zip › 4.png]
