# Supplementary material for: The stratification and prognostic importance of molecular and immune landscapes in clear cell renal cell carcinoma
Source: Front Oncol. 2023 Oct 2;13:1256720. doi: 10.3389/fonc.2023.1256720 (PMC10577421; doi:10.3389/fonc.2023.1256720)

	pvalue	Hazard ratio
GYG2	<0.001	1.051(1.030−1.071)
CHI3L2	<0.001	1.019(1.011−1.026)
PANX2	<0.001	1.036(1.018−1.054)
PPP2R2C	<0.001	1.089(1.060−1.119)
MOCOS	<0.001	1.060(1.042−1.078)
APOL1	<0.001	1.000(1.000−1.001)
PLTP	<0.001	1.001(1.001−1.001)
HAMP	<0.001	1.080(1.055−1.106)
DKK1	<0.001	1.016(1.009−1.023)
LIN7A	<0.001	0.981(0.972−0.989)
IL1R2	<0.001	1.001(1.001−1.002)
TREM1	<0.001	1.018(1.009−1.027)
C1QL1	<0.001	1.002(1.001−1.003)
GFPT2	<0.001	1.021(1.015−1.026)
PSAT1	<0.001	1.005(1.003−1.007)
AGTR1	<0.001	0.971(0.959−0.984)
CCNO	<0.001	1.050(1.027−1.074)
UCHL1	<0.001	1.005(1.003−1.007)
C1R	<0.001	1.002(1.001−1.003)
COL22A1	<0.001	1.073(1.048−1.099)
NMRAL2P	<0.001	1.095(1.057−1.136)
SLC6A19	<0.001	0.991(0.987−0.995)
IL20RB	<0.001	1.003(1.002−1.004)
FDCSP	<0.001	1.001(1.000−1.001)
C1S	<0.001	1.002(1.001−1.002)
TSKU	<0.001	1.005(1.003−1.007)
EFNA5	<0.001	1.009(1.005−1.012)
PRAME	<0.001	1.006(1.004−1.009)
SBSN	<0.001	1.016(1.008−1.023)
SP5	<0.001	1.061(1.034−1.089)
TPT1P5	<0.001	1.069(1.042−1.096)
MEG3	<0.001	1.056(1.033−1.079)
AL162586.1	<0.001	1.093(1.052−1.135)
SNTG2−AS1	<0.001	1.024(1.014−1.035)
LINC00894	<0.001	1.218(1.119−1.327)
SMKR1	<0.001	1.085(1.043−1.129)
AP001992.1	<0.001	1.128(1.063−1.196)
YJEFN3	<0.001	1.100(1.062−1.140)
AC005586.1	<0.001	1.079(1.049−1.110)
AC099343.2	<0.001	1.116(1.066−1.169)
MIR6819	<0.001	1.047(1.027−1.068)
AL079338.1	<0.001	1.013(1.008−1.018)

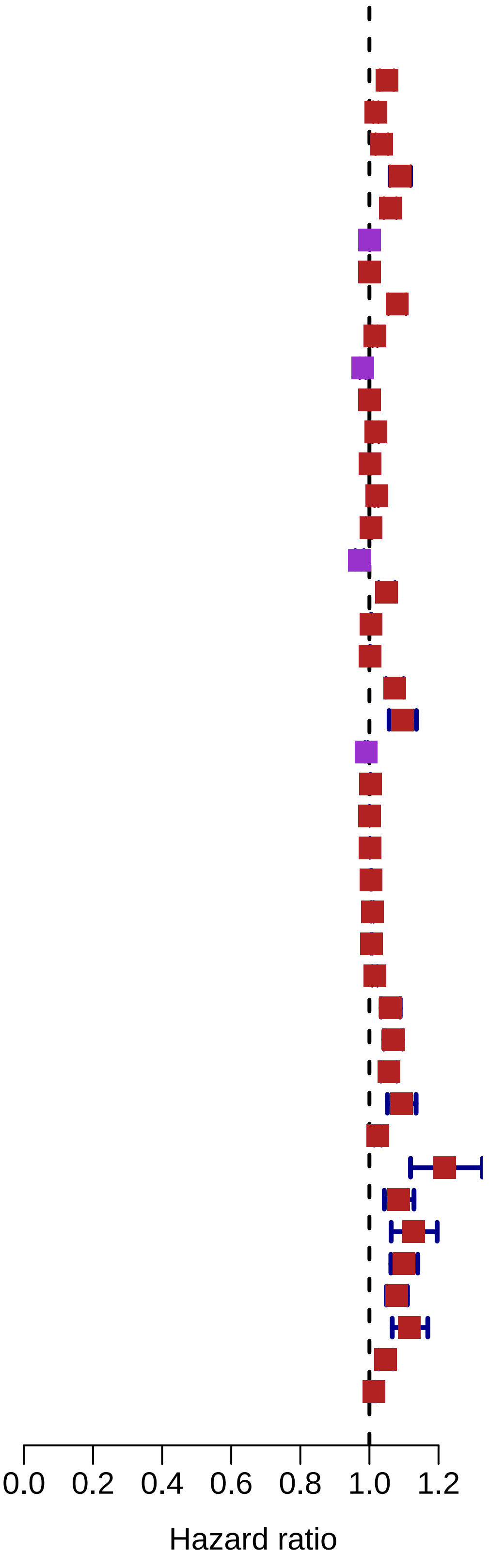

Supplement: Supplementary file 3 [file DataSheet_3.zip › forest.pdf]
