# Supplementary material for: The stratification and prognostic importance of molecular and immune landscapes in clear cell renal cell carcinoma
Source: Front Oncol. 2023 Oct 2;13:1256720. doi: 10.3389/fonc.2023.1256720 (PMC10577421; doi:10.3389/fonc.2023.1256720)
Supplement: Supplementary file 5 [file DataSheet_5.zip › Rplot.pdf]

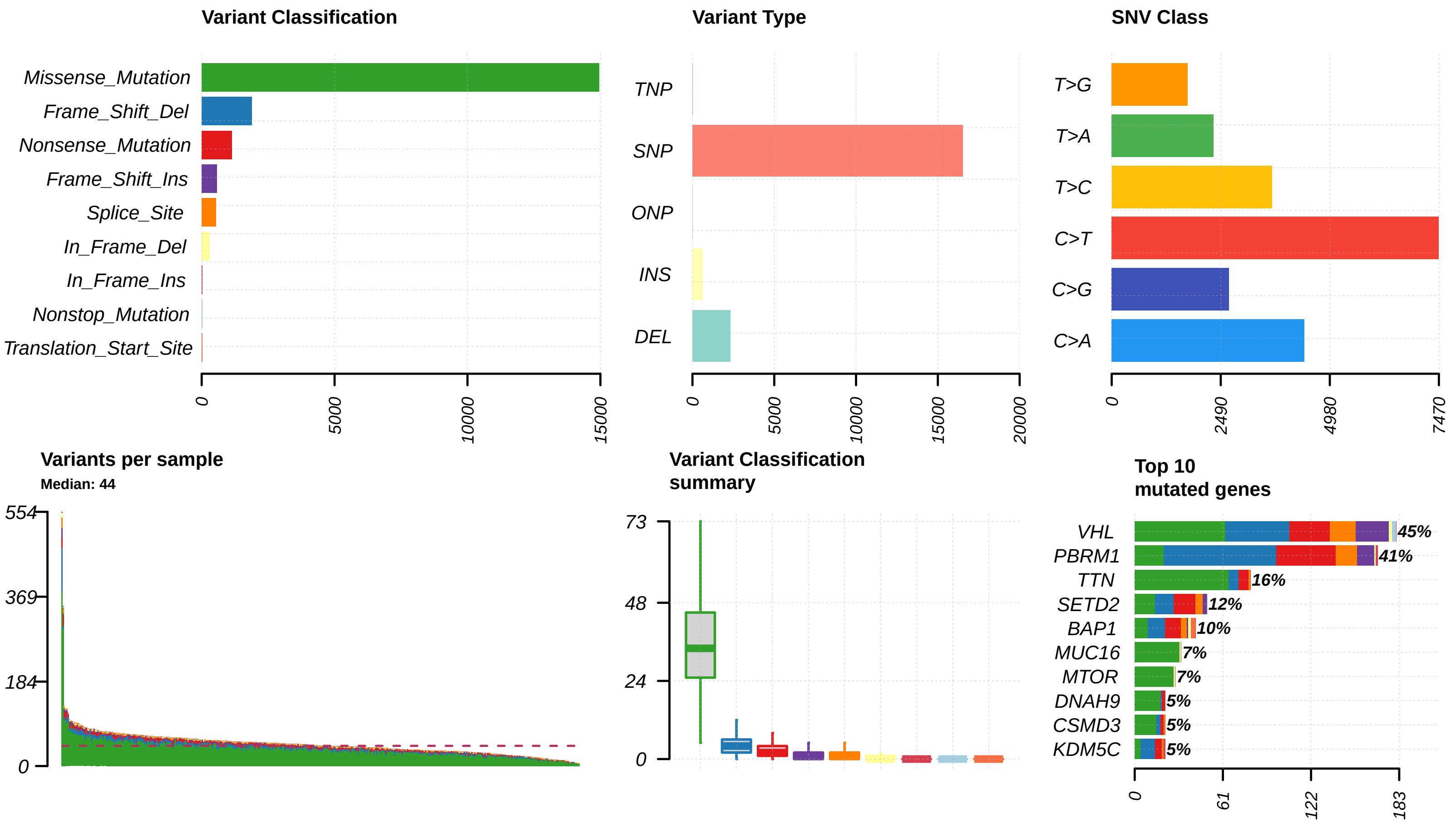
Variant Classification
Missense_Mutation
Frame_Shift_Del
Nonsense_Mutation
Frame_Shift_Ins
Splice_Site
In_Frame_Del
In_Frame_Ins
Nonstop_Mutation
Translation_Start_Site
0
5000
10000
15000
Variant Type
TNP
SNP
ONP
INS
DEL
0
5000
10000
15000
20000
SNV Class
T>G
T>A
T>C
C>T
C>G
C>A
0
2490
4980
7470
Variants per sample
Median: 44
554
369
184
0
Variant Classification summary
73
48
24
0
Top 10 mutated genes
VHL 45%
PBRM1 41%
TTN 16%
SETD2 12%
BAP1 10%
MUC16 7%
MTOR 7%
DNAH9 5%
CSMD3 5%
KDM5C 5%
0
61
122
183
