# Supplementary material for: The stratification and prognostic importance of molecular and immune landscapes in clear cell renal cell carcinoma
Source: Front Oncol. 2023 Oct 2;13:1256720. doi: 10.3389/fonc.2023.1256720 (PMC10577421; doi:10.3389/fonc.2023.1256720)

Altered in 326 (81.09%) of 402 samples.

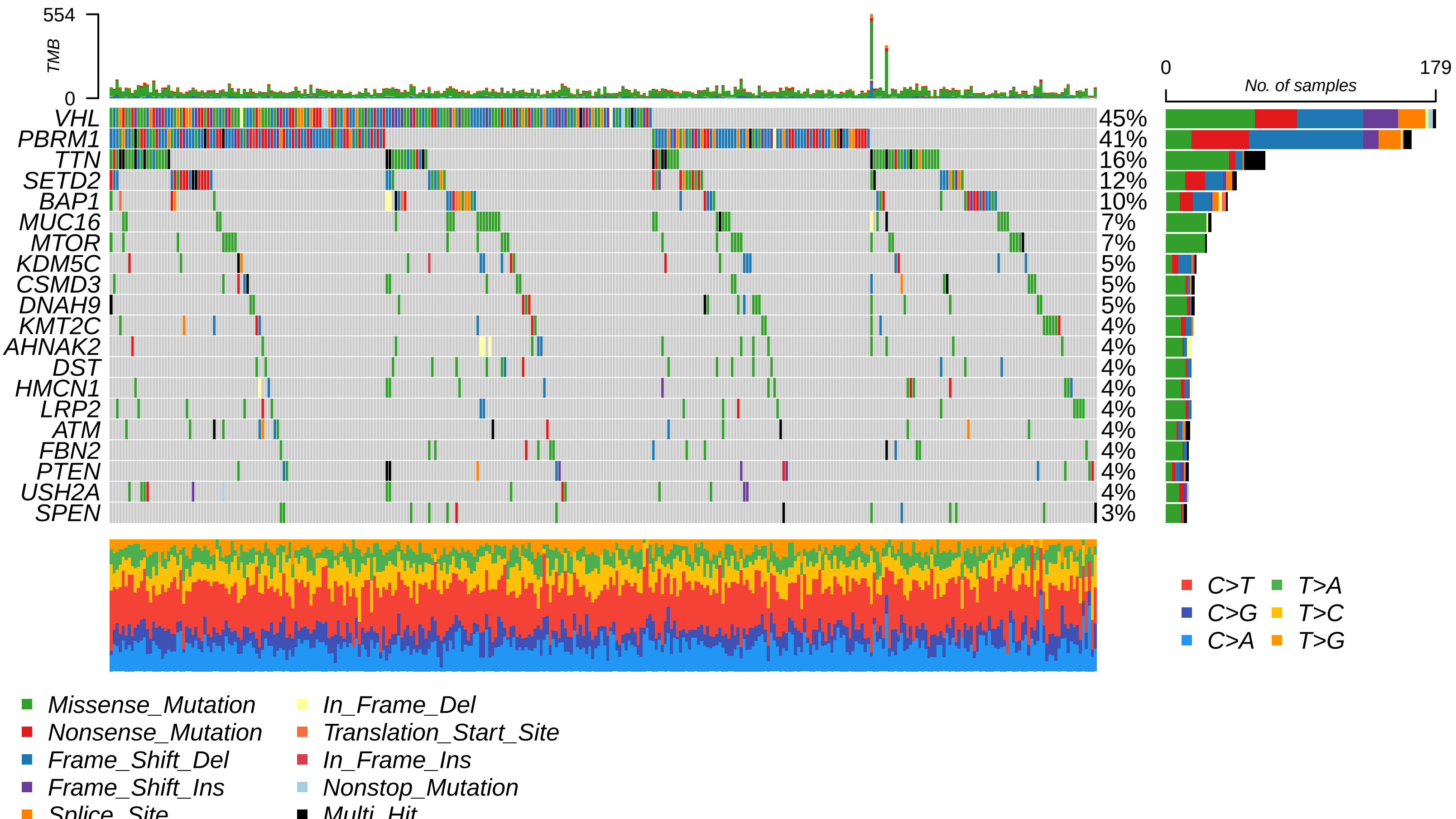

Supplement: Supplementary file 5 [file DataSheet_5.zip › Rplot01.pdf]
